# CAD hijacks STING to impair antitumor immunity and radiotherapy efficacy of colorectal cancer

**DOI:** 10.1038/s41419-025-07964-8

**Published:** 2025-08-23

**Authors:** Zhengkun Cai, Zeyuan Cheng, Lu Zhang, Yuhan Zhang, Jinming Shi, Jing Jin, Zeyun Mi, Zhiyong Yuan, Zhiqiang Wu

**Affiliations:** 1 Department of Radiation Oncology, Tianjin Medical University Cancer Institute & Hospital, Key Laboratory of Cancer Prevention and Therapy, National Clinical Research Center for Cancer, Tianjin’s Clinical Research Center for Cancer, Tianjin, China; 2 Department of Radiation Oncology, National Cancer Center/National Clinical Research Center for Cancer/Cancer Hospital, Chinese Academy of Medical Sciences and Peking Union Medical College, Beijing, China; 3 Department of Public Laboratory, Tianjin Medical University Cancer Institute & Hospital, Tianjin, China

**Keywords:** Translational research, Radiotherapy

## Abstract

Radiotherapy (RT)-elicited antitumor immunity serves as a pivotal mechanism in RT-mediated tumor control. The strategic integration of RT with immunotherapies, particularly immune checkpoint blockade (ICB), is revolutionizing cancer therapeutics, demonstrating remarkable clinical potential. In this context, identifying molecular targets to potentiate radioimmunotherapy (RIT) efficacy represents a critical research priority. Emerging as a central immunomodulatory axis, the cGAS/STING signaling pathway bridges DNA damage response with antitumor immunity, positioning itself as a prime therapeutic target for radiation sensitization. Our study unveils caspase-activated DNase (CAD) as a previously unrecognized suppressor of cGAS/STING signaling that governs radiosensitivity in colorectal cancer (CRC). CAD physically blocks STING dimerization and cGAMP binding through a nuclease-independent function, thereby compromising RT-induced STING activation and subsequent type I interferon (IFN-I) production. Functional analyses demonstrated that CAD ablation potentiates CD8^+^ T cell infiltration/activation within the tumor microenvironment and synergizes with anti-PD-1 immunotherapy upon radiation. Translational validation revealed clinical correlations between CAD overexpression in CRC specimens and suboptimal radiotherapy responses coupled with diminished intratumoral CD8^+^ T cell infiltration. Collectively, our findings establish CAD as a novel rheostat of cGAS-STING signaling and propose CAD inhibition as a promising combinatorial strategy to enhance RT and RIT efficacy in CRC.

## Introduction

Colorectal cancer (CRC) remains a leading cause of global cancer-related mortality, with persistent therapeutic challenges in advanced stages [1]. While radiotherapy (RT) serves as a cornerstone in multimodal CRC management, intrinsic radioresistance frequently compromises clinical outcomes [2–4]. Therefore, it is urgent to explore the molecular mechanism of radiotherapy resistance in CRC and to find novel potential strategies. The emerging paradigm of radioimmunotherapy (RIT) synergistically combines RT’s immunogenic potential with immune checkpoint inhibitors, yet mechanistic insights governing RIT efficacy remain incompletely characterized.

The abscopal effect [5], a systemic antitumor immune phenomenon triggered by localized irradiation, epitomizes radiation-induced immune activation. In addition to releasing abundant tumor-associated neoantigens, accumulated cytosolic DNA that triggering of IFN-Is signaling contributes to the onset of abscopal effect of radiotherapy. The cGAS-STING signaling has been well-demonstrated in sensing cytosolic DNA and provoking IFN-Is signaling as well as the subsequent antitumor immune response [6, 7]. cGAS catalyzes cyclic GMP-AMP (cGAMP) synthesis upon DNA sensing, activating STING’s conformational rearrangement and Golgi translocation [8]. At the Golgi apparatus, STING further recruits and activates TANK-binding kinase (TBK1) to phosphorylate itself as well as interferon regulatory factor 3 (IRF3). The phosphorylated IRF3 then translocates into the nucleus to transcribe IFN-Is and chemokines, establishing an immunogenic tumor microenvironment (TME) [9, 10]. Despite promising preclinical data, clinical translation of STING agonists faces limitations [11], suggesting undiscovered negative regulators within this pathway that may dictate therapeutic refractoriness. Illustrating the underlying mechanisms will provide novel strategies to improve efficacy of radiotherapy and immunotherapy.

Caspase-activated DNase (CAD), also known as DNA fragmentation factor subunit beta, is a well-known nuclease involved in nucleolysis and genome decomposition during sub-apoptotic caspase signaling [12, 13]. CAD is in complex with CAD endonuclease subunit and inhibitor of CAD (ICAD) in the resting state [14]. Upon activation of apoptosis, ICAD is proteolyzed and inactivated by Caspase-3, resulting in release of CAD. The freed CAD functions as a pair of molecular scissors to fragment DNA [12, 15]. Numerous research have reported the important role of CAD in regulating chromatin condensation and DNA fragmentation following apoptosis triggers in various models [16–18]. However, the role of CAD in cancer is less studied.

Herein, we identified CAD as a novel STING-interacting partner that constrains radiation-induced antitumor immunity. We demonstrate CAD’s nuclease-independent suppression of STING oligomerization and IFN-I production. CRISPR-mediated CAD ablation potentiates CD8^+^ T cell infiltration and synergizes with anti-PD-1 therapy in syngeneic models. Clinically, high expression of CAD correlates with radiotherapy resistance and immune-cold TME in CRC specimens. Our findings position CAD as a druggable rheostat of cGAS-STING signaling, proposing CAD inhibition as a strategy to overcome RT/RIT resistance in CRC.

## Materials and methods

### Patient specimens

Human tissues were obtained under the approval of the National Cancer Center/Cancer Hospital, Chinese Academy of Medical Sciences (Approval No. CH-GI-090). All participants provided informed written consent. The tissue samples were obtained from surgery patients with preoperative radiotherapy or postoperative radiotherapy at the Biobank of National Cancer Center/Cancer Hospital, Chinese Academy of Medical Sciences (CAMS). Twenty pre-treatment CRC tissues were used for IHC staining to analyze the expression of CAD and CD8.

### Cell culture

Human intestinal epithelial cell line NCM460, CRC cell lines, HCT116, HT29, SW480, H620, and mouse CRC cell line, MC38, were cultured in RPMI 1640 media (Gibco, Grand Island, USA). HEK293T cells were cultured in Dulbecco’s modified Eagle’s media (Gibco, Grand Island, USA). The media were supplemented with 10% fetal bovine serum (Gibco) and 1% penicillin-streptomycin (Gibco, Grand Island, USA). Cells were cultured at 37 °C in incubators containing 5% CO_2_. All cell lines were confirmed to be free of mycoplasma contamination and their identities were validated through short tandem repeat analysis.

### Plasmid constructs and transfection and stable cell line construction

Flag-tagged CAD, myc- or HA-Tagged cGAS were amplified from cDNA of HCT116 cells and inserted into pSin-EF2-IRES-puro lentiviral vector plasmid. Different types of tagged STING and mutants and mcherry-tagged TBK1 were derived plasmids reported previously [19]. Mouse Cad and Sting were amplified from cDNA of MC38 cells and cloned into the pSin-EF2-IRES-puro or pCDNA3.1 vector plasmid respectively. Human and mouse STING CCDS fragments were cloned into the pcDNA3.1 vector plasmid. Two guide RNA targeting *h*CAD and one targeting *m*Cad were designed and cloned into lentiCRISPRv2 plasmid for knockout of *h*CAD or *m*Cad. The sequences of the guide RNAs are listed in Supplemental Table 1. Transfection was performed using Lipofectamine 2000 (Invitrogen, Carlsbad, USA) according to the manufacturer’s instruction. The construction of stable cell lines was performed as described previously [20]. All the plasmids were verified through DNA sequencing.

### Bulk RNA-seq analysis

For RNA-seq data from HCT116 cells, total RNA was extracted using TRIzol (Invitrogen, Carlsbad, USA) and then sequenced using the Illumina NovaSeq 6000 platform (Illumina, San Diego, USA). The raw fastq data were processed using the fastp software (https://github.com/OpenGene/fastp) to obtain clean data. The read counts for each gene were calculated using featureCounts (v1.5.0-p3). Differential expression analysis was conducted using the DESeq2 R package (v1.36.0), and differentially expressed genes (DEGs) were identified based on the criteria of *P* adj < 0.05 and |fold change| ≥ 1.5. Gene Ontology (GO) enrichment analysis of DEGs and GSEA were implemented by the clusterProfiler R package (v4.4.4).

### Quantitative real-time PCR

RNA extractions were performed using the SPARK easy Cell RNA Rapid Extraction Kit (Shandong Sparkjade Biotechnology Co., Ltd., Shandong, China) according to the manufacturer’s protocol. cDNA synthesis was performed using the SPARK script II All-in-one RT SuperMix for qPCR (Shandong Sparkjade Biotechnology Co., Ltd., Shandong, China) and quantitative PCR reaction was monitored with PerfectStart® Green qPCR SuperMix (TransGen Biotech, Beijing, China) by the LC480 qPCR system (Roche, Mannheim, Germany). The 2^(−ΔΔCt)^ method was used for comparative Ct and GAPDH was used as a control. The primer sequences are listed in Supplemental Table 2.

### Dual-Luciferase reporter assay

5 × 10^4^ HCT116 cells or HT29 cells were seeded into each well of a 24-well plate and cultured overnight. 200 ng luciferase reporter plasmids were co-transfected with 5 ng pRL-TK plasmids (Promega, Madison, USA) into cells. The cells were treated with 8 Gy radiation 24 h after transfection. The luciferase activity was measured 24 h post RT using a Dual-Luciferase Reporter Kit (Promega, Madison, USA). The firefly luciferase activity was normalized to Renilla luciferase activity.

### Western blotting

Western blotting was performed as previously reported [21]. Nuclear and cytoplasmic extracts were prepared using the Nuclear and Cytoplasmic Protein Extraction Kit (Biyuntian, Beijing, China). Primary antibodies against CAD (1:500, #sc-374067, Santa Cruz Biotechnology, Santa Cruz, USA), Phospho-TBK1(Ser172) (1:1000, #5483, Cell Signaling Technology, Danvers, USA), TBK1 (1:1000, #38066, Cell Signaling Technology, Danvers, USA), Phospho-IRF3 (Ser396) (1:500, #29047, Cell Signaling Technology, Danvers, USA), IRF3(1:1000, #66670, Proteintech, Wuhan, China), Phospho-STING(Ser366)(1:500,#50907, Cell Signaling Technology, Danvers, USA), STING (1:1000, #13647, Cell Signaling Technology, Danvers, USA), cGAS(Asp175) (1:1000, #15102, Cell Signaling Technology, Danvers, USA), DYKDDDDK Tag (1:1000, #14793, Cell Signaling Technology, Danvers, USA), MYC-tag (1:1000, #16286, Proteintech, Wuhan, China), HA-Tag (1:1000, #3724, Cell Signaling Technology, Danvers, USA), GAPDH (1:4000, #60004, Proteintech, Wuhan, China), beta Tubulin (1:1000, #sc-166729, Santa Cruz Biotechnology, Santa Cruz, USA), mCherry (1:1000, #YM3132, Immunoway, USA), Lamin B1 (1:1000, #YM3036, Immunoway, USA) were used.

### Immunofluorescence microscopy

5 × 10^4^ HCT116 or HT29 cells were seeded on coverslips that were transfected with the plasmids or stimulated with radiation or cGAMP for the indicated time, then fixed in 4% paraformaldehyde for 15 min, permeabilized in 0.2% Trion X-100 for 15 min and blocked in 5% bovine serum albumin for 1 h at room temperature. Primary antibody against CAD (1:50, sc-#374067, Santa Cruz Biotechnology, Santa Cruz, USA), IRF3(1:100, #66670, Proteintech, Wuhan, China), Flag tag (1:500, #M20008, Abmart, Shanghai, China), MYC tag (1:1000, #16286, Proteintech, Wuhan, China) and Alexa Fluor 594 IgG, Alexa Fluor 488 IgG secondary antibodies (1:500, ZSGB-Bio, Beijing, China) were used. The nuclei were stained with DAPI (Sigma–Aldrich, St Louis, USA). Slides were mounted with ProLong Diamond Anti-fade reagent (Invitrogen, Carlsbad, USA). Immunofluorescence images were acquired under a Zeiss LSM 880 confocal microscope (Zeiss, Oberkochen, Germany). The fluorescence intensity of STING was quantified using ImageJ/Fiji.

### Co-immunoprecipitation assay

HEK293T, HCT116 and HT29 cells were harvested by ice-cold PBS and lysed with IP lysis buffer (25 mM HEPES, 150 mM NaCl, 1 mM EDTA, 1% NP-40, 10% Glycerol, pH 7.4) containing PMSF, at 4 °C for 1 h, then were pre-cleared with Protein A/G Magnetic Beads (Selleck, Houston, USA) for 1 h. Antibody against Flag (#M20008, Abmart, Shanghai, China), MYC (#60003, Proteintech, Wuhan, China), HA (#M20003, Abmart, Shanghai, China), STING (#13647, Cell Signaling Technology, Danvers, USA) and IgG (B900620, Proteintech, China) were added to the cell lysate for the antibody crosslink at 4 °C overnight with rotation. Then the cell lysates were incubated with Protein A/G Magnetic Beads (Selleck, Houston, USA) for 1 h. The beads were washed with lysis buffer for five times, and then were incubated with sample loading buffer and boiled for 10 min. The lysate samples were loaded on SDS-PAGE for further study.

### Mass spectrometry analysis

HCT116 cells that steadily overexpressed Flag-tag CAD were lysed with the lysis buffer, and the lysates were collected and incubated with anti-Flag immunological magnetic beads (Selleck, Houston, USA) at 4 °C overnight with rotation for crosslink. The beads were washed with IP buffer at least five times, eluted with 0.1 M glycine HCl (pH 3.0). The eluted samples were subjected to SDS-PAGE and silver staining analyses with Pierce Silver Stain Kit (Invitrogen, Carlsbad, USA). The stained gels were subjected to in-gel tryptic digestion. The resulting peptides were separated using reverse-phase liquid chromatography on an easy-nLC 1000 system (Thermo Fisher Scientific) and directly sprayed into a Q Exactive mass spectrometer (Thermo Fisher Scientific). The mass spectrometry data were analyzed using PD search engine (Thermo Fisher Scientific, v 3.0).

### Recombinant protein purification and GST pull-down assay

CCDS of STING or CAD were cloned into pGEX-6p1 vector plasmid and transformed the BL21 bacteria (TSC-E0z1, Tsingke Biotechnology, Beijing, China). The protein was induced by 0.1 mM IPTG at 16 °C overnight. Bacteria were spun down and lysed by ultrasonication in the BL21 bacteria lysate (1 M Tris-HCl, pH 7.5, 5 M NaCl, 0.2 mM PMSF, 1 mM DTT, and 1% Triton). Clear lysates were incubated with GST beads, and washed with bacteria lysate for 4 times. Proteins were eluted directly from the GST beads were or digested using PreScission enzyme (Biyuntian, Beijing, China) at 4 °C for at least 16 h and collected. Purified GST-Tagged CAD or STING then co-incubated with untagged STING or CAD, as well as the GST-conjugated beads at 4 °C overnight. Next day, the beads were spun down, washed, and the bound proteins were analyzed by western blotting assay.

### Molecular docking

The crystal structures of STING (4EMU) and CAD (1IBX) were obtained from the Protein Data Bank (https://www.rcsb.org/). The proteins were prepared with AutoDockTools-1.5.7. Protein-protein docking was performed using the Docking Web Server (GRAMM). The protein-protein surface interaction calculations and the protein-protein interaction figures were generated using PyMOL.

### In vitro 2′3′-cGAMP binding assay

The protein-2′3′-cGAMP-Cy5 conjugate reactions were set up in 1.5 mL centrifuge tubes. Added 2 µL of 2′3′-cGAMP-Cy5 (1 mM) (20318, AAT Bioquest, Pleasanton, USA), 5 µL reaction buffer (100 mM Tris, 500 mM KCl, and 10 mM DTT), and 0.5 µL of 200 mM EDTA (pH = 8), 40 µl of STING-GST recombinant protein and different concentrations of purified CAD protein, and the samples’ volume was increased to 800 µL by adding RIPA buffer (50 mM Tris HCl, 150 mM NaCl, 0.5% sodium deoxycholate, 1% IGEPAL). After precipitation overnight at 4 °C, beads were washed with RIPA buffer for 3 times. Finally, the bound proteins were eluted and subjected for western blotting analysis.

### Xenograft tumor model

C57BL/6J mice were purchased from the SPF Biotechnology Co., Ltd (Beijing, China). The 6-week-old female mice were randomized into the indicated groups. For Fig. 5A, 5 × 10^5^ MC38 sgVec and Cad knockout (Cad-KO) cells were subcutaneously implanted into the right outer thigh of each mouse. A dose of 8 Gy once was used when the tumor volumes reached around 150 mm^3^. To detect the percentage of CR (*n* = 6 per group), a dose of 10 Gy was used. For evaluating CAD mediating radiotherapy sensitivity via STING, mice (*n* = 5 per group) were treated with H-151(20 mg/kg, every 3 days, 3 times, S6652, Selleck, USA) or DMSO via intraperitoneal injection when the tumor volumes reached ~150 mm^3^. One day after the first H-151 inhibitor treatment, mice received 8 Gy radiotherapy once. For tumor rechallenge experiments, mice (*n* = 5 per group) were injected with 5 × 10^5^ MC38 sgVec and Cad-KO cells and taken a radiation of 10 Gy once to lead CR of the tumors. Around 20 days after IR treatment, the Cad-KO group reached CR. MC38 sgVec cells were rechallenged on the contralateral flank of the Cad-KO group mice or the naïve mice with 5 × 10^5^ at day 40. For the detection of abscopal effects of radiation, 5 × 10^5^ MC38 sgVec and Cad-KO cells were injected into the right outer thigh of mice (*n* = 5 per group) as the primary tumor and 3 × 10^5^ MC38 sgVec cells were injected into the corresponding left flank of the same mice as the abscopal tumor. The primary tumors were irradiated with 8 Gy. For combined therapy analysis, mice (*n* = 5 per group) were irradiated at day 8 with 8 Gy and intraperitoneal injected with anti-PD-1 (αPD-1) monoclonal or isotype IgG2a antibody (10 mg/kg, every 3 days for 3 times, BP0146, BP0089, Bioxcell, USA). The endpoint for experiments was the tumor volume reaching around 2000 mm^3^. Tumors were measured with calipers every 2 days, and mice conditions were monitored every day. The tumor volume was calculated by the following formula: Volume = 0.5 × length × width^2^. After humane euthanasia of the mice, the tumors were excised, weighed, and fixed in 4% formalin for further analysis. All animal experimental protocols were conducted in accordance with the NIH Guide for the Care and Use of Laboratory Animals (National Academies Press, 2011) and approved by the Laboratory Animal Ethics Committee of Tianjin Medical University Cancer Institute and Hospital (Approval No. AE2024004).

### IHC analysis

Immunohistochemistry was performed as described previously [21]. Antibodies against CAD (1:50, sc-374067, Santa Cruz Biotechnology, USA), Phospho-TBK1 (Ser172) (1:100, #5483, Cell Signaling Technology, USA), Phospho-IRF-3 (Ser396) (1:100, #29047, Cell Signaling Technology, USA), Ki67 (1:100, 27309, Proteintech, China), Granzyme B (1:100, A2557, Abclonal, China), CD8 (1:1000, 66868, Proteintech, China) were used. The images were acquired at 100× and/or 400× magnification using an OLYMPUS BX61 microscope (Olympus, Tokyo, Japan). ImageJ software was used to quantify CAD and CD8 IHC staining. The percentage of positively stained area was calculated by using a color deconvolution for separating the staining components in at least five fields each sample. The results were presented as percentage of average optical density = Integrated Density/Area.

### Flow cytometry

Tumor tissue was harvested from C57BL/6J model mice into RPMI 1640 medium containing 1% collagenase IV (Solarbio, Beijing, China) and then incubated at 37 °C for 30 min. The tumors were dissociated mechanically through 70-µm cell filters (BD Biosciences, San Jose, USA) to generate single-cell suspensions. Samples were analyzed using the anti-mouse specific antibodies obtained from BioLegend. Anti-CD45 (30-F11), anti-TCR-β (H57-597), anti-CD8a (53-6.7), anti-Granzyme B (QA16A02), anti-IFN-γ (XMG1.2), anti-CD11b (M1/70), anti-Gr1 (RB6-8C5), anti-CD206 (C068C2), anti-CD86 (GL-1), anti-CD11c (N418), anti-MHCII (M5/114.15.2), anti-F4/80 (BM8) were used. Samples were acquired on CytoFLEX LX (Beckman, USA) and analyzed by CytExpert.

### ELISA

Concentrations of cGAMP in cells were measured by 2′3′-cGAMP ELISA Kit (501700, Cayman Chemical) according to the manufacturer’s instructions. Ifn-β in mice serum were measured by Mouse *Ifn-β* (Interferon Beta) ELISA Kit (Elabscience) according to the manufacturer’s instructions. The optical density (OD) value was detected at a wavelength of 450 nm by a microplate reader (BioTek, Winooski, USA).

### T Cell-killing assay

Human or mouse peripheral blood mononuclear cells (PBMCs) were separated from whole blood by a density gradient centrifugation method using Ficoll (LTS1077-1, Tianjin Haoyang Biological Manufacture; 7211011, Dakewe Biotech Company Limited, China), and stimulated with anti-CD3 antibody (1 μg/ml, #317326, #100339, Biolegend, San Diego, USA), anti-CD28 antibody (1 μg/ml, #302934, #122021, Biolegend, San Diego, USA), and interleukin 2 (30 ng/ml, #200-02-50UG, Gibco, Grand Island, USA; #503706, Biolegend, San Diego, USA) for 48 h. The activated PBMCs were further co-cultured with the irradiated HCT116 or MC38 cells at a ratio of 1:5 in 96-well plates for 24 h. The cells were finally fixed with methanol and stained with 1% crystal violet.

### SPR measurement

SPR experiments were performed using Biacore 8 K instrument (GE Healthcare). CAD proteins were captured on sensor chip CM5. STING proteins with increasing concentrations were injected into the protein surface for 2 min, dissociated for 2 min in running buffer. Equilibrium constants were calculated by Biacore 8 K evaluation software.

### Statistics

GraphPad Prism version 8.0 and SPSS Statistics version 25 were used to carry out the statistical analysis. Two-tailed Student’s *t*-test, one-way analysis of variance (ANOVA), Two-way RANOVA with Tukey’s test, Pearson correlation, or Spearman correlation analysis were performed for statistical comparisons. Survival curves were plotted by the Kaplan–Meier method and compared using the log-rank test. Survival data were evaluated using univariate and multivariate Cox regression analyses. Unless indicated otherwise, all results were presented as mean ± SD. *P* value less than 0.05 is considered statistically significant (*P < 0.05, **P < 0.01, ***P < 0.001, ns: not significant).

## Results

### CAD directly interacts with STING

**Fig. 1:**
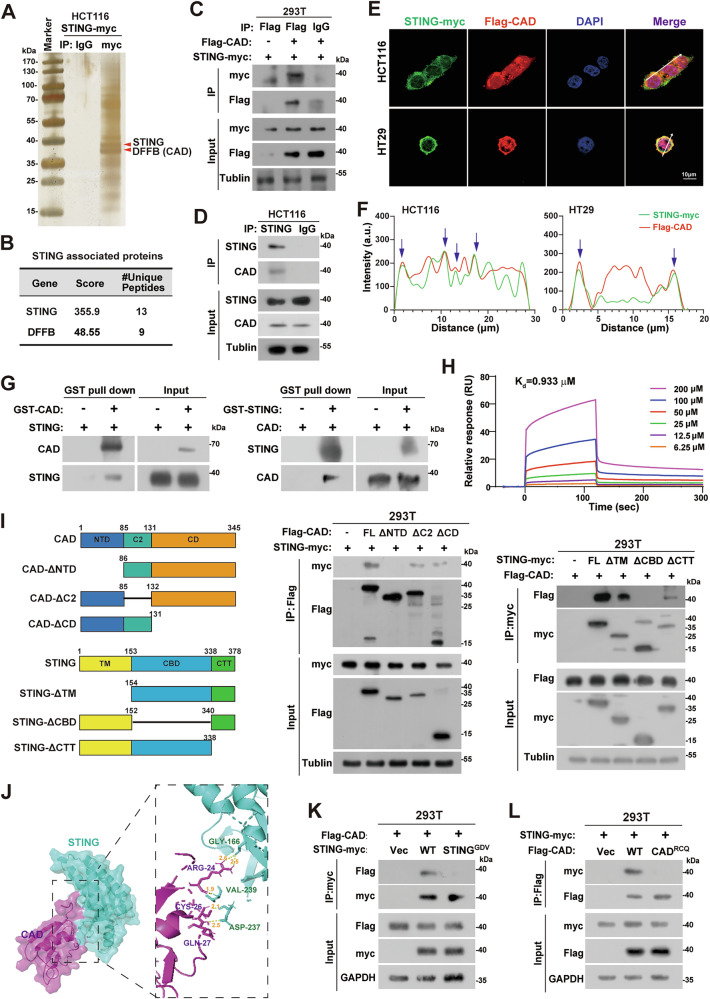
CAD interacts with STING. **A** Representative image of silver staining of proteins co-immunoprecipitated with CAD. Red arrows indicated as the bands of CAD and STING protein. **B** CAD associated proteins determined by mass-spectrometry analysis. **C**, **D** Immunoprecipitation and western blotting analyzing the interaction between exogenous (**C**) or endogenous (**D**) CAD and STING in the indicated cells. **E**, **F** Representative confocal fluorescence microscopy images (**E**) and colocalization analyses (**F**) of CAD and STING. Scale bars, 10 μm. Blue arrows indicate the colocalization sites. **G** GST pull-down assays analyzing the interaction between CAD and STING. **H** SPR assay analyzing the binding affinity of STING protein with CAD protein. **I** Schematic representation of human STING and CAD mutants (upper). Immunoprecipitation and western blotting analyses of the interaction between STING mutants and CAD mutants (lower). **J** Molecular docking analyzing the interface between CAD and STING. **K** Immunoprecipitation and western blotting analyses of the interaction between CAD and the STING^GDV^ mutant. **L** Immunoprecipitation and western blotting analyses of the interaction between STING and the CAD^RCQ^ mutant.

STING plays a central role in the cGAS/STING/IFN-Is signaling. We thus performed protein immunoprecipitation combined with mass spectrometry analysis to identify novel factors that might modulate the activity of cGAS-STING signaling via interacting with STING. Interestingly, the CAD protein was identified as a potential STING-interacting protein (Fig. 1A, B and Supplemental Fig. 1A). A series of protein co-immunoprecipitation (Co-IP) and western blotting analyses confirmed that both the exogenous and endogenous CAD and STING interacted with each other (Fig. 1C, D). Similar results were obtained in MC38 cells of the mouse Cad and Sting protein (Supplemental Fig. 1B, C). Additionally, immunofluorescence staining results showed that CAD colocalized with STING (Fig. 1E, F). GST pull-down assay further demonstrated the direct interaction of the two proteins (Fig. 1G and Supplemental Fig. 1D). Surface plasmon resonance (SPR) was applied to analyze the binding kinetics of CAD-STING and confirmed the strong affinity (Kd = 0.993 μM, Fig. 1H). Although STING co-immunoprecipitated with CAD as well as TBK1 and IRF3 (Supplemental Fig. 1E), it seemed that they formed different complexes, as there was no detectable interaction between CAD and cGAS, TBK1, or IRF3 (Supplemental Fig. 1F).

Moreover, we mapped the interaction domains and sites between CAD and STING. Results showed that the cytoplasmic ligand-binding domain (CBD, amino acid 154–338) of STING and the ICAD-binding N-terminal domain (NTD, amino acid 6–85) of CAD are essential for the interaction between CAD and STING (Fig. 1I). Molecular docking analysis found that Arg24, Cys26, and Gln27 of the CAD NTD domain form hydrogen bonds with Gly166, Asp237, and Val239 of the STING CBD domain, respectively (Fig. 1J). Single point mutation of these sites of both CAD and STING to Ala alone did not interrupt the interaction between CAD and STING (Supplemental Fig. 1G). Therefore, we mutated the 3 sites simultaneously of CAD (R24A, C26A, and Q27A, named CAD^RCQ^) or STING (G166R, D237A, and V239F, named STING^GDV^), respectively. The result showed that neither the STING^GDV^ mutant interacts with CAD nor the CAD^RCQ^ mutant interacts with STING (Fig. 1K, L). These results proved that the R24, C26, and Q27 in the NTD domain of the CAD and G166, D237, and V239 in the CBD domain of STING are the interacting sites between them.

### Depleting CAD activates STING/IRF3/IFN-Is signaling

**Fig. 2:**
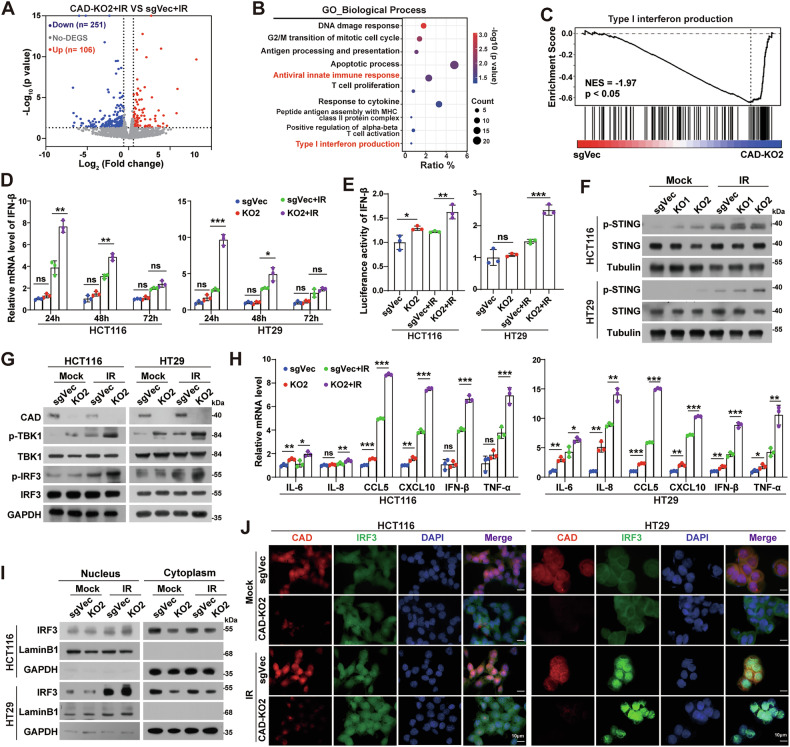
Depleting CAD activates STING/IRF3/IFN-Is signaling. **A** Volcano plot of differentially expressed genes between CAD-KO and sgVec control HCT116 cells at 24 h after 8 Gy irradiation. **B** Gene Ontology (GO) term enrichment analysis of DEGs. **C** GSEA analysis of the Type I interferon production in the CAD-KO2 HCT116 cells. **D** qPCR analyses of IFN-β mRNA in indicated cells at 24, 48, and 72 h after irradiation. **E** Activity of the IFN-β luciferase reporter in sgVec control and CAD-KO cells at 24 h after irradiation. **F** Western blotting analyses of phospho-STING (p-STING) and STING levels in indicated cells at 24 h after irradiation. Tubulin served as a loading control. **G** Western blotting analyses of CAD, phospho-TBK1 (p-TBK1), TBK1, phospho-IRF3 (p-IRF3), and IRF3 levels in the indicated cells at 24 h after irradiation. GAPDH served as a loading control. **H** qPCR analyses of IL-6, IL-8, CCL5, CXCL10, IFN-β, and TNF-α mRNA in indicated cells at 24 h after irradiation. **I** Western blotting analyses of the IRF3 level in the nuclear and cytoplasmic fractions of the indicated cells at 24 h after irradiation. Lamin B1 and GAPDH served as nuclear and cytoplasmic loading control, respectively. **J** Representative images of immunofluorescence staining of IRF3 in indicated cells at 24 h after irradiation. Scale bars, 10 μm. Error bars represent mean ± SD from 3 independent experiments. *P* values were determined using two-tailed, unpaired *t*-test between different groups (**D**, **E** and **H**). *, *P* < 0.05; **, *P* < 0.01; ***, *P* < 0.001; ns not significant.

After evaluating the expression of CAD in a variety of CRC cells, HCT116 and HT29, as well as the mouse CRC cell line MC38, were selected to construct CAD overexpression and/or knockout (KO) stable cell lines for further study (Supplemental Fig. 2A–C). RNA-seq analysis showed that knockout of CAD resulted in 106 upregulated and 256 downregulated genes after IR treatment (Fig. 2A). GO analysis (Fig. 2B), as well as the gene set enrichment assay (Fig. 2C), of these DEGs revealed significant enrichment in terms related to the innate immune response and IFN-Is production in CAD-KO2 cells. These findings suggested that CAD might regulate IFN-Is production and innate immunity induced by IR. Indeed, qPCR analyses showed that the expression of IFN-β was significantly induced in CAD knockout cells after IR, particularly at 24 h post-treatment (Fig. 2D). Moreover, luciferase reporter assay showed that depleting CAD increased, while overexpression of CAD decreased, the IFN-β reporter activity in both HCT116 and HT29 cells after IR treatment (Fig. 2E and Supplemental Fig. 2D). These findings proved that CAD negatively regulates the expression of IFN-β.

It is well known that IR can activate cGAS-STING signaling to induce IFN-Is and inflammatory cytokines [14]. To verify whether CAD modulates the expression of IFN-β through cGAS-STING signaling, we first analyzed the expression of phospho-STING (p-STING). The results showed that knockout of CAD significantly enhanced the expression of p-STING after IR (Fig. 2F). In addition, the levels of phospho-TBK1 (p-TBK1) and phospho-IRF3 (p-IRF3) were also increased in CAD depleted cells (Fig. 2G and Supplemental Fig. 2E, F), but decreased in CAD overexpressing cells (Supplemental Fig. 2G), after IR treatment. Quantitative PCR analyses showed that the expressions of CCL5, CXCL10, IL-6, IL-8, IFN-β, and TNF-α, which are downstream genes of STING/IRF3 signaling, were significantly enhanced in CAD-KO cells compared to control groups (Fig. 2H and Supplemental Fig. 2H), but impaired in CAD overexpressing cells (Supplemental Fig. 2I), upon IR treatment. Similar results were observed under the situation that STING was activated by cGAMP (Supplemental Fig. 2J–L). Moreover, subcellular fragments analysis and immunofluorescence assays revealed that inhibition of CAD promoted the accumulation of IRF3 in the nucleus after IR (Fig. 2I, J and Supplemental Fig. 2M). Overall, these data demonstrated that CAD negatively regulates cGAS-STING signaling and depleting CAD enhances IFN-Is after IR treatment.

### CAD inhibits dimerization/polymerization, cGAMP-binding, and Golgi apparatus translocation of STING to block the pathway

**Fig. 3:**
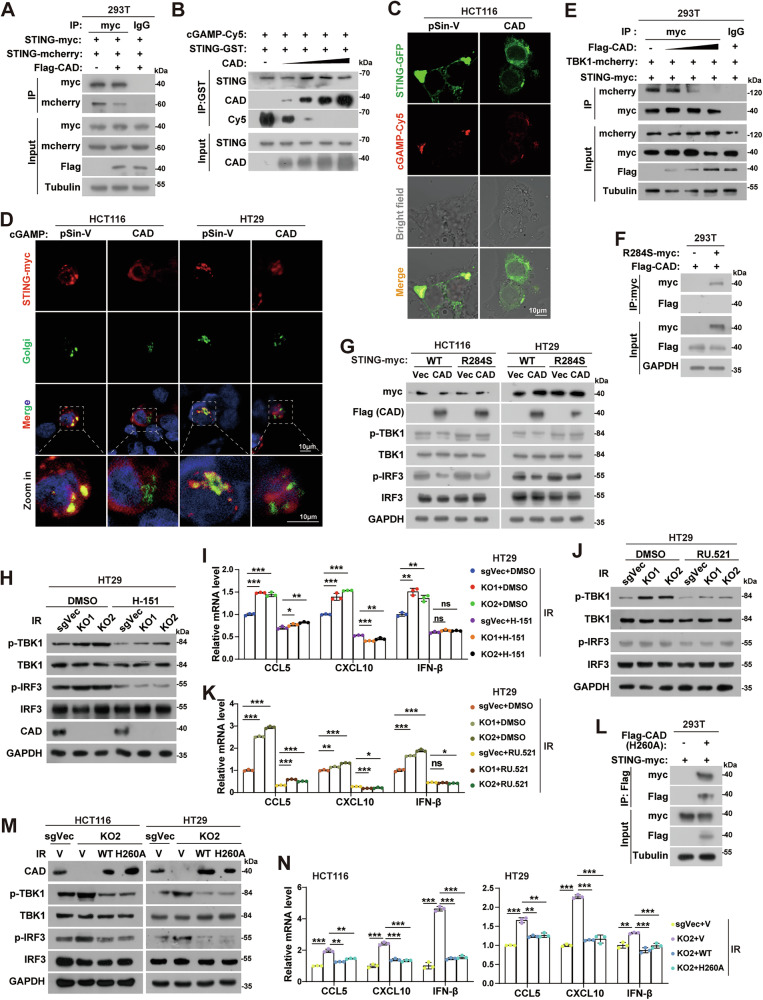
CAD impairs STING dimerization, cGAMP-binding, and Golgi apparatus translocation independent on its nuclease activity. **A** Immunoprecipitation and western blotting analyses of the effects of CAD on STING dimerization/oligomerization in HEK293T cells. **B** Immunoprecipitation and western blotting analyses of the effect of CAD on the interaction between STING and cGAMP in HEK293T cells transfected with different amount of CAD. **C** Representative confocal fluorescence microscopy images of STING-EGFP and cGAMP-Cy5 in indicated cells. Scale bars, 10 μm. **D** Representative confocal fluorescence microscopy images of STING and Golgi apparatus in indicated cells stimulated with cGAMP. Scale bars, 10 μm. **E** Immunoprecipitation and western blotting analyses of the effect of CAD on the interaction between STING and TBK1. **F** Immunoprecipitation and western blotting analyses of the interaction between CAD and the STING (R284S) mutant in HEK293T cells. **G** Western blotting analyses of p-TBK1, TBK1, p-IRF3, and IRF3 levels in HCT116 and HT29 vector control or Flag-CAD overexpressed cells that transfected with myc-tagged wild type (WT) STING or the R284S mutants at 24 h after irradiation. **H** Western blotting analyses of p-TBK1, TBK1, p-IRF3, and IRF3 levels in indicated cells at 24 h after treatment of irradiation plus DMSO or H-151 treatment. **I** qPCR analyses of CCL5, CXCL10, and IFN-β mRNA in indicated cells at 24 h after irradiation with DMSO or H-151 treatment. **J** Western blotting analyses of p-TBK1, TBK1, p-IRF3, and IRF3 levels in indicated cells at 24 h after treatment of irradiation plus DMSO or RU.521 treatment. **K** qPCR analyses of CCL5, CXCL10, and IFN-β mRNA in indicated cells at 24 h after irradiation with DMSO or RU.521 treatment. **L** Immunoprecipitation and western blotting analyses of the interaction between STING and CAD (H260A) mutant in HEK293T cells. **M** Western blotting analyses of p-TBK1, TBK1, p-IRF3, and IRF3 levels in control cells and the CAD-KO2 cells that reintroduced the wild type (WT) or nuclease activity dead mutant (H260A) of CAD at 24 h after irradiation. **N** qPCR analyses of CCL5, CXCL10, and IFN-β mRNA in indicated cells at 24 h after irradiation. Error bars represent mean ± SD. *P* values were determined using two-tailed, unpaired *t*-test (**I**, **K**, and **N**). *, *P* < 0.05; **, *P* < 0.01; ***, *P* < 0.001; ns not significant.

STING is an endoplasmic-reticulum membrane protein that forms a butterfly-shaped dimer in the inactive state [22, 23]. Binding to cGAMP leads to a conformational change of the cytoplasmic ligand-binding domain (CBD), which results in the formation of the STING polymers and subsequent translocation to Golgi apparatus for full activation [22]. It drew our attention that G166 and D237 are the interacting sites of STING dimer and V239 of STING directly interacts with the guanine base of cGAMP [23–25], which are the sites mediating STING and CAD interaction identified in this study (Fig. 1I, J). Therefore, we hypothesized that CAD inhibited the dimerization/polymerization and binding to cGAMP of STING. Indeed, immunoprecipitation combined with Western blotting assays showed that overexpression of CAD dramatically reduced the interaction between STING molecules (Fig. 3A) and that between STING and cGAMP in a dose-dependent manner (Fig. 3B). Immunofluorescence assays showed that overexpression of CAD reduced colocalization of STING and cGAMP as well as the perinuclear STING aggregates (Fig. 3C). Moreover, less STING translocated to the Golgi apparatus was observed in the CAD overexpressing cells upon cGAMP stimulation (Fig. 3D). Additionally, CAD significantly impaired the recruitment of TBK1 to STING in a dose-dependent manner (Fig. 3E). To further validate these findings, we constructed the reported SAVI-causing STING mutants, R284S, which is constitutively activated in cells [26, 27]. We found that CAD could not interact with STING-R284S (Fig. 3F) or inhibit the signaling induced by STING-R284S, as there was no differences of the expression of p-TBK1 and p-IRF3 in STING-R284S transfected cells no matter with CAD overexpressed or not (Fig. 3G), which might because of the constitutive polymerization of STING R284S [28].

H-151, an inhibitor of STING, significantly abolished the provoking effects of CAD depletion combined with IR on the expression of p-TBK1 and p-IRF3 (Fig. 3H and Supplemental Fig. 3A) and the downstream genes (Fig. 3I and Supplemental Fig. 3B) in HT29, HCT116, and MC38 cells (Supplemental Fig. 3C, D). The cGAS inhibitor, RU.521, got the similar effects as that of H-151 (Fig. 3J, K and Supplemental Fig. 3E, F). However, CAD did not interact with cGAS (Supplemental Fig. 2E) or impair the formation of cGAS dimer (Supplemental Fig. 3G) and the cGAMP level (Supplemental Fig. 3H). These results suggested that CAD specifically acts on STING to impair STING/IFN-Is signaling.

Since CAD works primarily as an endonuclease, we wondered whether its nuclease activity is essential for inhibiting STING. Co-IP assay showed that the nuclease activity dead mutant CAD-H260A [29] interacted with STING normally (Fig. 3L). Introduction of the CAD-H260A into CAD-KO cells blocked the expression of p-TBK1 and p-IRF3 and the downstream genes provoked by knockout of CAD combined with IR to a similar level as that of the WT CAD (Fig. 3M, N and Supplemental Fig. 3I, J). Additional CAD nuclease-dead mutants of *h*CAD-D259A and *m*Cad-D262A showed similar effects as *h*CAD-H260A (Supplemental Fig. 3K–N). These findings demonstrated that CAD impaired STING signaling independent on its nuclease activity.

### ICAD reverses the inhibitory role of CAD on STING signaling

**Fig. 4:**
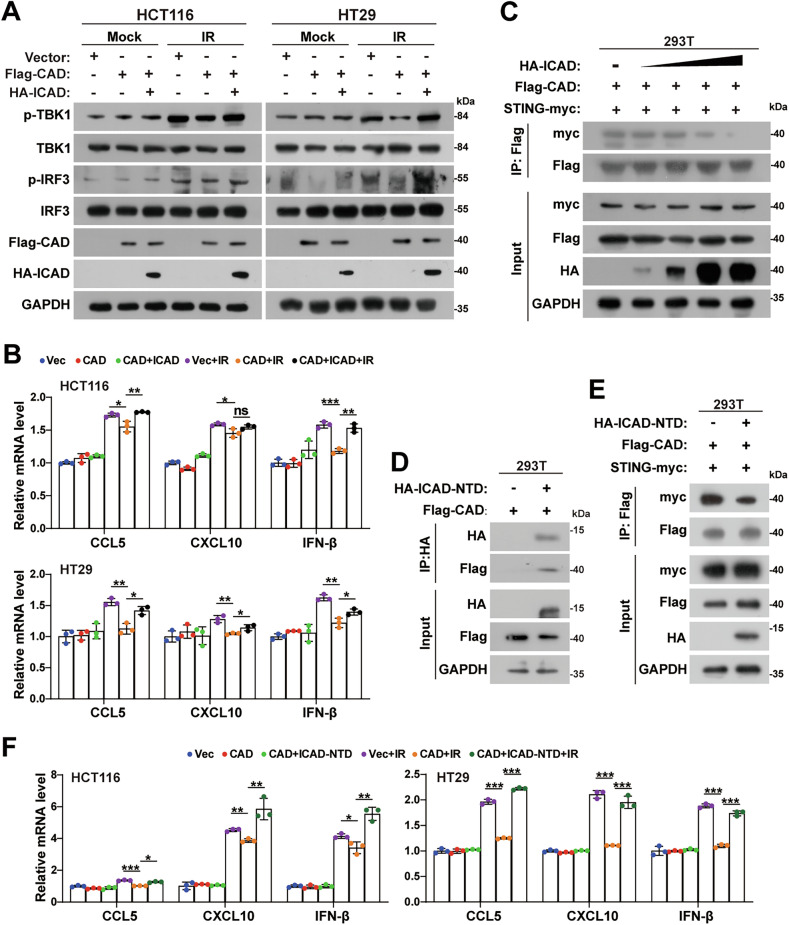
ICAD reverses the inhibitory role of CAD on STING signaling. **A** Western blotting analyses of p-TBK1, TBK1, p-IRF3, and IRF3 levels in HCT116 and HT29 cells after ectopic overexpression of CAD and inhibitor of CAD (ICAD). **B** qPCR analyses of CCL5, CXCL10, and IFN-β mRNA in indicated cells at 24 h after irradiation. Error bars represent mean ± SD. **C** Immunoprecipitation and western blotting analyses of the interaction between STING and CAD upon overexpressing ICAD in HEK293T cells. **D** Immunoprecipitation and western blotting analyses of the interaction between STING and ICAD in HEK293T cells. **E** Immunoprecipitation and western blotting analyses of the interaction between STING and CAD upon overexpressing ICAD-NTD in HEK293T cells. **F** qPCR analyses of CCL5, CXCL10, and IFN-β mRNA in indicated cells at 24 h after irradiation. Error bars represent mean ± SD. *P* values were determined using two-tailed, unpaired *t*-test (**B** and **F**). *, *P* < 0.05; **, *P* < 0.01; ***, *P* < 0.001.

Under physiological condition, ICAD complexes with CAD to prevent CAD dimerization and cleaving DNA [30]. Thus, we hypothesized that ICAD would affect the intervention of CAD in STING pathway. Indeed, western blotting assays showed that overexpressing ICAD rescued the expression of p-TBK1 and p-IRF3 upon IR treatment, which were inhibited by CAD overexpression (Fig. 4A). Real-time PCR assays revealed that ICAD abolished CAD-triggered reduction of CCL5, CXCL10, and IFN-β after radiotherapy (Fig. 4B). These results proved that ICAD could reverse the inhibition role of CAD on STING signaling activation. Interestingly, though the interaction between CAD and ICAD was constantly detected (Supplemental Fig. 4A), no interaction between ICAD and STING was identified in human or mouse cells (Supplemental Fig. 4B, C). Hence, we speculated that ICAD enhanced STING signaling by competing with STING for binding CAD. As expected, overexpressing ICAD significantly impaired the interaction between CAD and STING in a dose-dependent manner (Fig. 4C). Previous studies have shown that ICAD bound with CAD via its NTD domain [31], then we evaluated whether the NTD domain of ICAD got the potential to reverse the effects of CAD on STING. Co-IP assay confirmed the interaction of ICAD-NTD and CAD (Fig. 4D) and revealed that ICAD-NTD did reduce CAD binding with STING (Fig. 4E). Quantitative PCR analyses showed that ICAD-NTD also rescued the expression of typical downstream genes of STING after radiotherapy (Fig. 4F).

### Inhibition of CAD enhances radiosensitivity of CRC in vivo through STING pathway

**Fig. 5:**
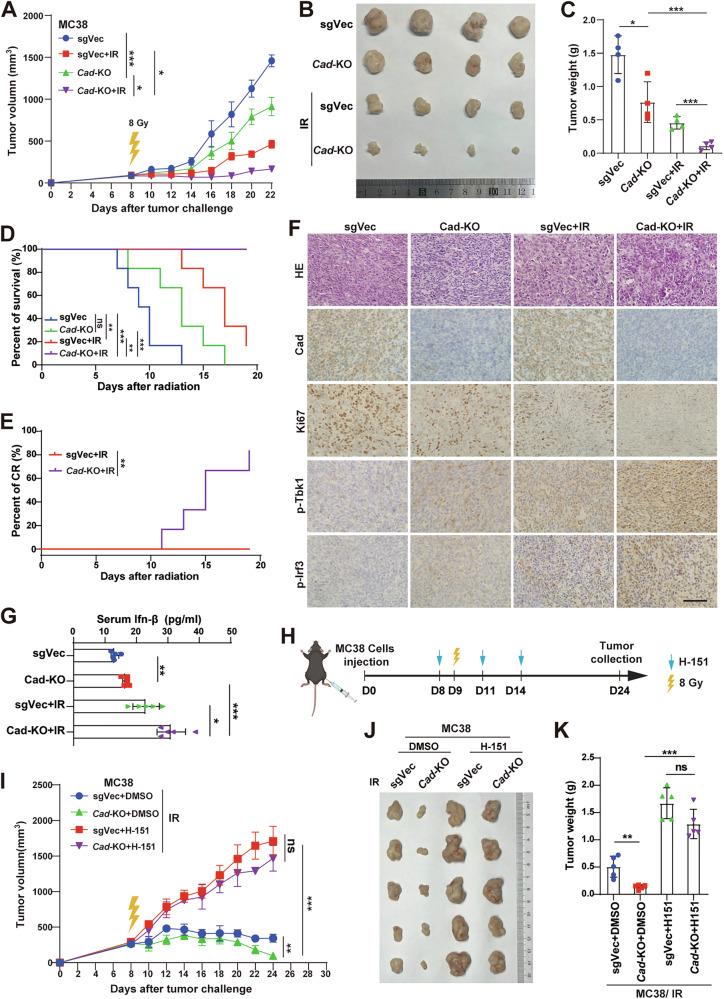
Inhibition of CAD enhances radiosensitivity of CRC in vivo through STING pathway. **A** Growth curve of the xenografts (*n* = 4 for each group) from the indicated MC38 cells irradiated with 8 Gy or not. **B** Representative images of tumors from mice in each group. **C** Quantification of tumor weights. **D** Kaplan–Meier survival analysis for mice (*n* = 6 per group) with injected cells after 10 Gy irradiation treatment at the tumor site or not treated. **E** Kaplan–Meier analysis of tumor complete response (CR) for mice (*n* = 6 per group) with injected cells after treatment of 10 Gy irradiation. **F** Representative images of H&E staining and IHC staining of Cad, p-Tbk1, p-Irf3, and Ki67 of the indicated group of tumors. Scale bars, 100 μm. **G** Serum Ifn-β level of different groups mice analyzed by ELISA. **H** Schematic diagrams of subcutaneous tumor models and the treatment. **I** Growth curve of the xenografts (*n* = 6 per group) from MC38 cells with the indicated treatments. **J** Representative images of tumors from mice in each group. **K** Quantification of tumor weights. Error bars represent mean ± SD. *P* values were determined using 1-way ANOVA with Tukey’s multiple comparison test (**C**, **G**, and **K**), Kaplan–Meier method with log-rank test (**D** and **E**) or Two-way RANOVA with Tukey’s test (**A** and **I**). *, *P* < 0.05; **, *P* < 0.01; ***, *P* < 0.001; ns not significant.

We next evaluated whether depleting CAD could radiosensitize CRC in vivo. We implanted Vector control (sgVec) or Cad-knockout (Cad-KO) MC38 cells on the flanks of C57BL/6 mice, and then monitored tumor growth after local radiation treatment of 8 Gy. The results showed that knockout of Cad alone could reduce tumor growth. While depleting Cad combining with radiotherapy dramatically impaired tumor growth further compared with the control (Fig. 5A–C). In addition, the survival rate of mice (Fig. 5D) and complete response rate of tumors (Fig. 5E) were much higher in the Cad-KO group than that of the control group after RT. IHC staining assay showed that the expression of Ki67 substantially reduced in tumors of the Cad-KO combined with RT group (Fig. 5F), further proved the poor proliferation of this group of tumors. However, phosphorylated Tbk1 and Irf3 expressions in tumors (Fig. 5F) and the serum Ifn-β level (Fig. 5G) were significantly upregulated in the Cad-KO combined with RT group. This indicated that depleting CAD highly potentiated IR-induced activation of STING signaling in vivo. To verify the activation of STING account for tumor regression caused by Cad-KO after radiotherapy, we used H-151 to block STING activation in vivo (Fig. 5H). The results showed that H-151 dramatically abolished the antitumor effects of depleting Cad combined with RT (Fig. 5I–K). These results suggested that inhibition of CAD could enhance radiosensitivity of CRC in vivo, which relies on the activation of cGAS-STING signaling.

### Inhibition of CAD promotes radiation-induced antitumor immunity and boosts radioimmunotherapy

**Fig. 6:**
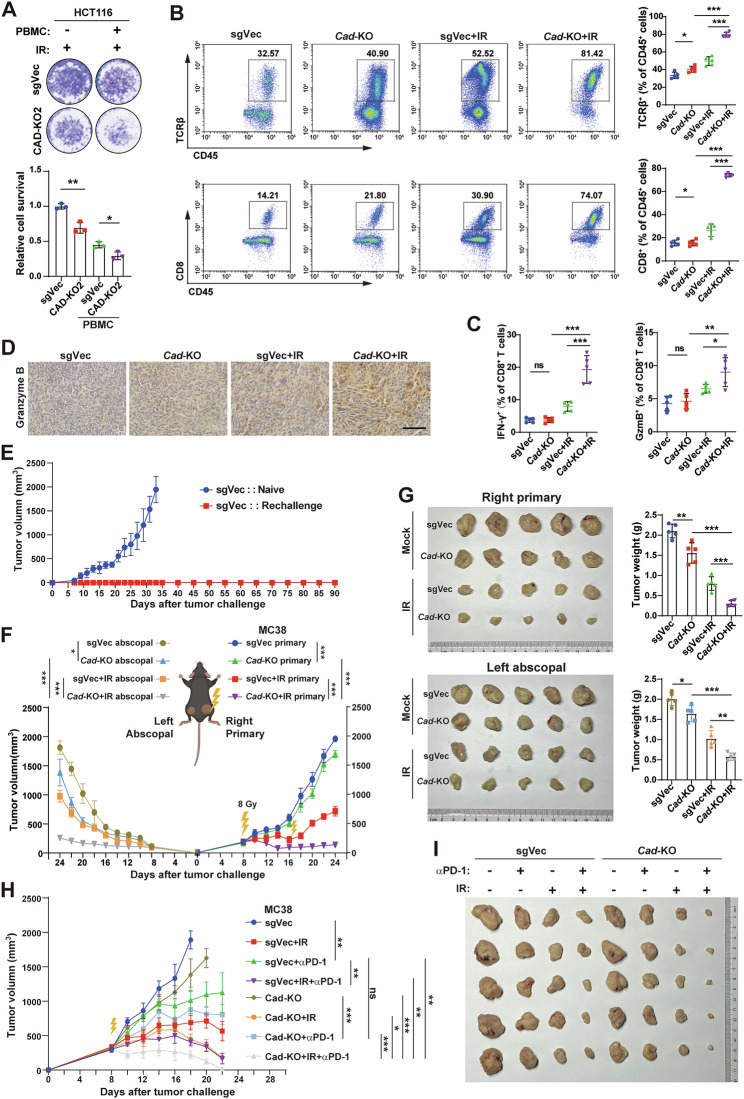
Inhibition of CAD promotes radiation-induced antitumor immunity and boosts radioimmunotherapy. **A** T cell killing efficiency of the indicated HCT116 cells treated with irradiation. Each spot intensity represents the live cancer cell quantity, and the relative fold ratio of surviving cells is shown in the lower panel. **B** Representative flow cytometry analyses (left) and quantification (right) of T cells (TCRβ^+^ cells, upper panel) and CD8^+^ T cells (lower panel) among CD45^+^ cells infiltrating MC38 tumors (*n* = 5 per group). **C** Quantification of Ifn-γ^+^ and Granzyme B^+^ cells among intratumoral CD8^+^ T cells. **D** Representative pictures of IHC staining of Granzyme B of the indicated groups of tumors. Scale bars, 100 μm. **E** Rechallenge growth curves of MC38 sgVec tumors in treatment naïve mice and the mice that reached complete remission after *Cad*-KO cells injection and irradiation. **F** Growth curve of the unirradiated abscopal tumors (MC38 sgVec cells, left flank) and the irradiated primary tumors (the indicated cells, right flank) in mice (*n* = 5 per group). **G** Representative images of tumors from all the mice in each group (left panel) and the quantification of tumor weights (right panel). **H** Growth curve of the xenografts from MC38 cells with the indicated treatments. (*n* = 5 per group). **I** Representative images of tumors from all the mice in each group. Error bars represent mean ± SD. *P* values were determined using two-tailed, unpaired *t*-test (**A**) one-way ANOVA with Tukey’s multiple comparison test (**B**, **C**, and **G**) or Two-way RANOVA with Tukey’s test (**F** and **H**). *, *P* < 0.05; **, *P* < 0.01; ***, *P* < 0.001; ns not significant.

STING/IFN-Is signaling has been demonstrated promoting antitumor immunity [6]. Hence, we hypothesized that depleting CAD enhanced IR-induced antitumor immunity. Firstly, we performed a T cell killing assay in which different groups of cells were irradiated and treated with or without PBMCs. The results showed that PBMCs had a remarkable killing effect on the cells in the CAD-KO group (Fig. 6A and Supplemental Fig. 5A). Then, we measured the proportion and function of CD45^+^TCRβ^+^ and CD8^+^ T cells of the tumors from different groups of mice by flow cytometry. Gating strategy for flow cytometry was shown in Supplemental Fig. 5B. We found that the amount of CD8^+^ T cell infiltration was significantly increased (Fig. 6B) and the expressions of Granzyme B and Ifn-γ were upregulated in CD8^+^ T cells in the combination treatment group (Fig. 6C). Consistently, IHC staining of the tumor tissues from Fig. 5B showed that the expression of Granzyme B was the highest in the IR treated Cad-KO group (Fig. 6D). Interestingly, we also found that the percentage of intratumoral DC cells (CD86^+^MHCII^+^) was also increased, but the MDSC cells (CD11B^+^Gr1^+^) was decreased, in the combination treatment group (Supplemental Fig. 5C, D).

To solidify the finding that inhibition of CAD enhanced IR-induced antitumor immunity, we performed tumor cell rechallenge and abscopal tumor experiments. The results showed that the mice that had achieved complete remission against MC38 (Cad-KO) tumors resistant to the re-attack of MC38 (sgVec) cells, while the naïve group developed tumors quickly (Fig. 6E). Moreover, depleting CAD significantly delayed the growth of both primary and abscopal tumors compared to control groups (Fig. 6F, G). IHC analysis revealed highest level of CD8^+^ cells in the abscopal tumors from the IR treated Cad-KO group (Supplemental Fig. 5E), proving the important effect of immune infiltration on inhibiting abscopal tumors. These results suggested that inhibition of CAD induced adaptive immunity and generated immunological memory against the tumor after radiotherapy.

Recently, multiple clinical trials showed that radiotherapy plus ICB therapy (radioimmunotherapy, RIT) achieved remarkable efficacy in different types of tumor [20, 32, 33] and has revolutionized the therapeutic guidelines. According to our findings, we proposed that depleting CAD could enhance the efficacy of RIT and performed experiment as Supplemental Fig. 5F. Results showed that RIT (radiotherapy combined with anti-PD-1) did dramatically impair tumor growth. Moreover, depleting CAD further enhanced the antitumor efficacy of RIT, leading to almost complete remission of the tumors (Fig. 6H, I). This indicated that CAD might serve as a potential target to enhance the efficacy of RIT for CRC.

### CAD is upregulated and negatively correlates with prognosis, radiotherapy response, and CD8^+^ cell infiltration in CRC

**Fig. 7:**
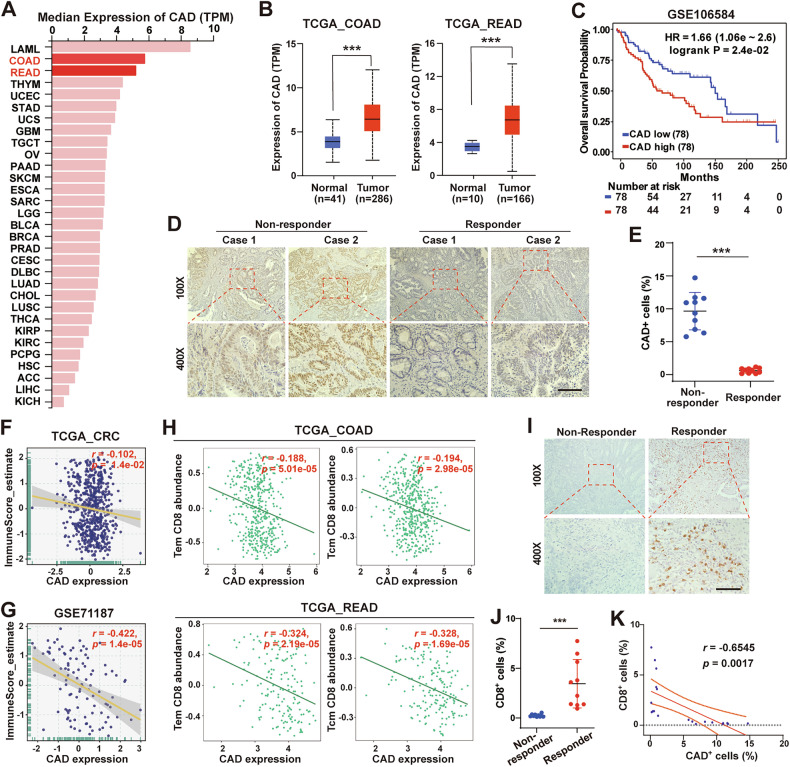
CAD is upregulated and negatively correlates with prognosis, radiotherapy response and CD8^+^ cell infiltration in CRC. **A** The expression profile of CAD across human cancers determined by the GEPIA website (http://gepia.cancer-pku.cn/about.html). **B** The expression of CAD in TCGA_COAD and TCGA_READ samples. **C** Kaplan–Meier analysis of overall survival (OS) in CRC patients from GEO/GSE106584 dataset. **D**, **E** Representative images (**D**) and quantification (**E**) of IHC staining for CAD in rectal cancer tissues from radiotherapy non-response (Non-Responder) and response (Responder) patients. Scale bars, 100 μm. **F**, **G** Correlation analyses between CAD expression and Immune score from the TCGA-CRC dataset (**F**) and GEO/GSE71187 dataset (**G**). *p* and *r* values were calculated by Pearson’s correlation test. **H** Correlation analyses between CAD expression and CD8^+^ T cells abundance of TCGA_COAD and TCGA_READ samples on TSIDB platform (http://cis.hku.hk/TISIDB/index.php). *p* and *r* values were calculated using Spearman correlation test. **I**, **J** Representative images (**I**) and quantification (**J**) of IHC staining of CD8 in rectal cancer tissues. Scale bars, 100 μm. **K** Correlation between the percent of CAD^+^ cells and that of CD8^+^ cells detected by IHC staining. *P* values were determined using Mann–Whitney test (**B**), Kaplan–Meier method with log-rank test (**C**), unpaired *t*-test (**E** and **J**) or *f*-test (**K**). *, *P* < 0.05; **, *P* < 0.01; ***, *P* < 0.001.

Lastly, we explored the expression and clinical relevance of CAD in CRC. Analysis from the GEPIA database (http://gepia.cancer-pku.cn/) showed that CAD is highly expressed in CRC (colon adenocarcinoma (COAD) and rectum adenocarcinoma (READ)) compared with most other types of cancer (Fig. 7A). Data from the UALCAN database (https://ualcan.path.uab.edu/analysis.html) showed significantly increased CAD expression in CRC tissues compared to the normal ones (Fig. 7B). Kaplan–Meier survival analysis of GEO/GSE106584 dataset showed that high CAD expression was associated with poor overall survival (OS) (*p* = 2.4e-2, HR = 1.66, 95% CI = 1.06–2.60) (Fig. 7C). Then, we evaluated the effects of CAD expression on radiotherapy response in a cohort of CRC patients. IHC staining analysis showed that the expression of CAD in the radiotherapy-resistant group (Non-responder) was significantly higher than that in the sensitive group (Responder) (Fig. 7D, E). These data suggested the important role of CAD in CRC and that high level of CAD is a poor prognosis factor of CRC and correlates with radioresistance.

Further, we analyzed the association between immune cell infiltration and CAD expression. Analyses showed that the expression of CAD negatively correlated with immune scores performed from the BEST platform in the TCGA-CRC (Fig. 7F) and GEO/GSE71187 (Fig. 7G) datasets. Consistently, evidence from the TISIDB platform also showed that CAD level was negatively correlated with both the infiltration of effector memory (Tem) and central memory CD8^+^ T cells (Tcm) (Fig. 7H) in the TCGA-COAD and TCGA-READ datasets. Moreover, we evaluated the infiltration of CD8^+^ T cells in CRC patient specimens by IHC assay (Fig. 7I). We found that the percentage of CD8^+^ cells was significantly higher in the responder group and negatively correlated with the expression of CAD (Fig. 7J, K). Our results suggested that the expression of CAD could predict immune infiltration and radiotherapy response in CRC, indicating that CAD may be a potential therapeutic target for CRC.

## Discussion

**Fig. 8:**
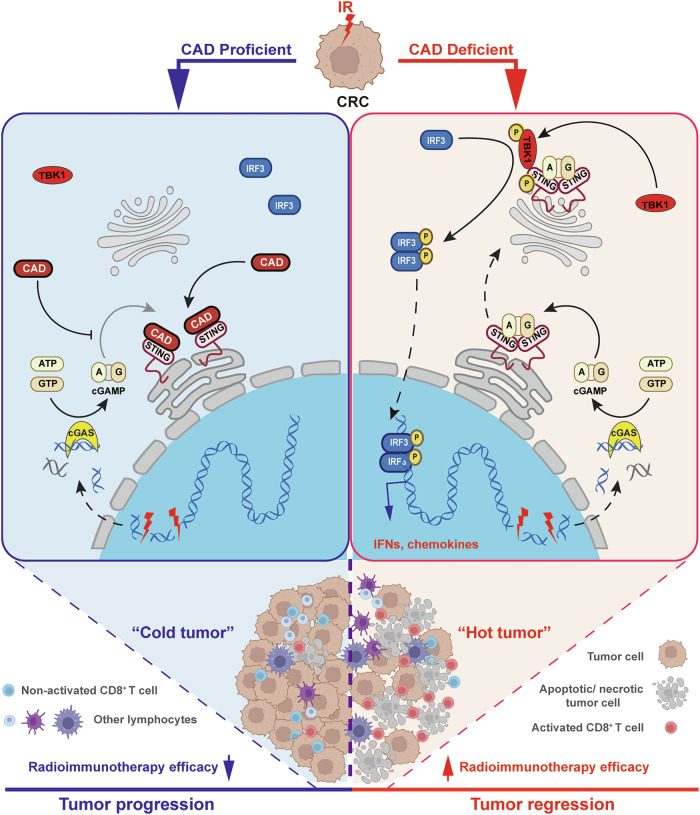
Schematic illustration of how CAD restraining cGAS-STING signaling to modulate radioimmunotherapy of CRC.

Based on the above-mentioned findings, CAD proved to be a key molecule inhibiting RT-elicited antitumor immunity through impairing cGAS/STING signaling. We revealed that CAD directly interacts with STING to block STING dimerization/polymerization and binding to cGAMP, resulting in retention of STING at endoplasmic reticulum (ER), reduction of IFN-Is and chemokines and the subsequent less infiltrated lymphocytes in tumor. Thus, silencing CAD enhanced RT-induced antitumor immunity and boosted RIT (Fig. 8).

More and more studies indicate that, in addition to breaking down DNA double strand to kill cancer cells directly, antitumor immunity-provoking is an important aspect of RT-induced tumor shrinking systemically. Clinical trials proved that RT combined with immunotherapy greatly improved the efficacy and prognosis of patients [20, 32, 33]. Increasing evidence showed that the cGAS/STING pathway is involved in RT-induced antitumor immunity and modulating the efficacy of RT-based therapy [34–37]. Nevertheless, several mechanisms are exploited by tumor cells or stromal cells to hinder the activation and signal transduction of the pathway at different levels, including enhancing DNA repair efficiency [38], and the regulation of the stability [37], localization [36], and activation [39] of key components in the cGAS/STING pathway. In this study, we revealed that CAD negatively regulates cGAS/STING signaling through a novel mechanism. We found CAD interacting with STING and identified G166, D237, and V239 in the CBD domain of STING as the interacting sites. Interestingly and importantly, G166 and D237 are the sites accounts for STING dimerization and V239 is the site of STING that interacts with cGAMP [23–25]. Hence, our results showed that CAD impaired the ability of dimerization and binding with cGAMP of STING, thus inhibiting the activation of the pathway and the subsequent antitumor immunity.

Currently, STING agonists, 2′3′-cGAMP and its synthetic analogues, showed great translational potential for clinical treatment of cancer in vitro studies. However, the clinical response is not satisfactory. Here, our results showed that CAD could block STING interacting with cGAMP (Supplementary Fig. 4J–L), which we propose to be the direct reason accounting for the unsatisfactory results of these clinical trials. Our results indicated that impairing CAD would advance the translational research of STING agonists. However, as the important role of CAD in cellular apoptosis, simply depleting CAD might reduce its effects on tumor suppression because of impaired apoptosis. Hence, it will kill two birds with one stone if we block the interaction between CAD and STING and not disturbing its role in apoptosis. Interestingly, our result revealed that the NTD domain of ICAD could be the stone. Our data showed that ICAD-NTD could liberate STING from CAD to allow activation of STING/IFN-Is signaling (Fig. 5E, F). According to previous report [31], the ICAD-NTD peptide is highly possible also releasing CAD from ICAD to forward apoptosis. Developing a peptide drug of ICAD-NTD for translational usage sounds promising. Previously, we delivered nuclear acids for in vivo cure of cancer in animal models by nanoparticles [40, 41]. We will take effort to develop ICAD-NTD based nanomedicine in the future. However, several challenges [42] for in vivo delivery needs to be overcomed and there is a long way to go.

CAD is the first identified nuclease fragmenting DNA upon apoptotic cell death [12, 43]. Although DNA fragmentation is a hallmark and thought to be the final step of cellular apoptosis, cell autonomous DNA fragmentation is not absolutely required for apoptosis execution [44]. In accordance with these findings, knockout or inhibition of CAD results in resistance to apoptotic DNA fragmentation but with little impact on overall cellular apoptosis [16, 45, 46]. Hence, DNA fragmentation might be just a by-product for overactivated CAD. This suggested that there is some other function of the nuclease activity of CAD or the function that independent on its nuclease activity. It has been reported that transient DNA strand break formation fosters gene expression in living cells [47, 48]. As expected, another work from Brian et al. revealed that CAD-inflicted DNA lesions enhanced the expression of critical genes to promote cell differentiation [49]. These findings suggested the importance of the nuclease activity of CAD in cell differentiation. Additionally, in the current study, we uncovered a novel function of CAD that independent on its nuclease activity (Fig. 4L–N). We proved that CAD could serve as a scaffold protein to bind with STING directly, leading to abolishment of STING/IFN-Is signaling. To our knowledge, these findings have not been reported before.

In the past years, both tumor-inhibiting and promoting roles of CAD had been reported. Bin Yan et al. showed that CAD null mice were susceptibility to radiation-induced carcinogenesis due to decreased genomic stability, indicating the tumor suppression role of CAD [45]. Nevertheless, a research from the same group demonstrated that CAD-associated self-inflicted DNA double strand breaks are important in sustaining the stemness of patient-derived glioma cells, and knockout of CAD impaired tumorigenic abilities of cancer cells [50]. Similarly, Brian et al. revealed that CAD-inflicted DNA lesions after IR provided more time for repair of IR-induced DNA damage, which enhances cancer cell survival and renders radioresistance [51]. The opposite function of CAD on carcinogenesis might because of different types of cancer. CAD was reported to be downregulated in various types of tumors, but with an exception of CRC [46, 52, 53]. Consistently, in the current study we found that CAD is upregulated in CRC. In addition, we further revealed that CAD is a poor prognosis factor for CRC patients, as high levels of CAD correlated with short overall survival time and radioresistance. In vitro and in vivo assays proved that depleting CAD delayed tumor progression and enhanced efficacy of RT and RIT. Hence, our results highlighted the tumor-promoting role of CAD in CRC. However, because of the relative hard accessibility of pre-treatment CRC tissues, only 20 CRC samples had been collected for clinical validation. This weakened the statistical power and is a limitation of our study.

In conclusion, our study revealed a novel mechanism that CAD inhibits STING dimerization and binding with cGAMP to impair RT-induced IFN-Is production and antitumor immunity. Our findings highlighted that CAD could be a promising target for enhancing the efficacy of RT and RIT of CRC.

## Supplementary information

Supplemental materials

Supplemental Figure 1

Supplemental Figure 2

Supplemental Figure 3

Supplemental Figure 4

Supplemental Figure 5

Uncropped western blots

## Data Availability

The bulk RNA sequencing dataset in this study has been deposited to the NCBI Gene Expression Omnibus database and the accession number is GEO: GSE283230 (https://www.ncbi.nlm.nih.gov/geo/query/acc.cgi?acc=GSE283230). The gene expression data of TCGA COAD and TCGA READ used in the study are available at TCGA website (https://portal.gdc.cancer.gov/). The data of GSE106584 and GSE71187 are available at the GEO database (https://www.ncbi.nlm.nih.gov/geo/). Any data used in this study that are not included in the paper or supplementary files can be made available upon request from the corresponding author.
