# Supplementary material for: CAD hijacks STING to impair antitumor immunity and radiotherapy efficacy of colorectal cancer
Source: Cell Death Dis. 2025 Aug 23;16(1):641. doi: 10.1038/s41419-025-07964-8 (PMC12375107; doi:10.1038/s41419-025-07964-8)
Supplement: Supplementary file 1 — Supplemental materials [file 41419_2025_7964_MOESM1_ESM.docx]

**Supplemental Tables**

**Supplemental Table 1. Target sequences for knockout of CAD by CRISPR-Cas9 system**

| **Name** | **Target sequence** |
| --- | --- |
| CAD-KO1 | GTGTTCCCGACAACGCCGAGC |
| CAD-KO2 | GTGCCTGTACGAGGATGGCA |
| Cad-KO | TCCCAACGACGCTGAGCTCC |

**Supplemental Table 2. Sequences of real-time PCR primers**

| **Primer name** | **Sequence** |
| --- | --- |
| CCL5_Fwd | CCAGCAGTCGTCTTTGTCAC |
| CCL5_Rev | CTCTGGGTTGGCACACACTT |
| CXCL10_Fwd | GTGGCATTCAAGGAGTACCTC |
| CXCL10_Rev | GTGGCATTCAAGGAGTACCTC |
| IFN-β_Fwd | GCTTGGATTCCTACAAAGAAGCA |
| IFN-β_Rev | ATAGATGGTCAATGCGGCGTC |
| IL-6_Fwd | AGACAGCCACTCACCTCTTCAG |
| IL-6_Rev | TTCTGCCAGTGCCTCTTTGCTG |
| IL-8_Fwd | TGATTTCTGCAGCTCTGTGTG |
| IL-8_Rev | TCTGTGTTGGCGCAGTGTGG |
| TNF-α_Fwd | AGGGACCTCTCTCTAATCAGC |
| TNF-α_Rev | TCTCAGCTCCACGCCATTGG |
| GAPDH_Fwd | CGGAGTCAACGGATTTGGTC |
| GAPDH_Rev | GACAAGCTTCCCGTTCTCAG |
| Ccl5_Fwd | CCAAGTGCTGCCGTCATTTTC |
| Ccl5_Rev | CCAAGTGCTGCCGTCATTTTC |
| Cxcl10_Fwd | CGATGACGGGCCAGTGAGAATG |
| Cxcl10_Rev | TCAACACGTGGGCAGGATAGGCT |
| Ifn-β_Fwd | AGATCAACCTCACCTACAGG |
| Ifn-β_Rev | TCAGAAACACTGTCTGCTGG |
| Il-6_Fwd | TCTGCAAGAGACTTCCATCCAGTTGC |
| Il-6_Rev | AGCCTCCGACTTGTGAAGTGGT |
| Il-8_Fwd | CCATGGCACATTCTGTTCAAA |
| Il-8_Rev | GCCCATCAGAGGCAAGGA |
| Tnf-α_Fwd | ATCCGCGACGTGGAACTG |
| Tnf-α_Rev | ACCGCCTGGAGTTCTGGAA |
| Gapdh_Fwd | CTCCACTCACGGCAAATTCAAC |
| Gapdh_Rev | GTAGACTCCACGACATACTCAGC |

**Supplemental Figure legends**

**Supplemental Figure 1. CAD interacts with STING but not the other proteins of cGAS-STING pathway.** (**A**) Representative image of DFFB (CAD) peptide identified by MS. (**B-C**) Immunoprecipitation and western blotting analyzing the interaction between exogenous (**B**) or endogenous (**C**) Cad and Sting in the MC38 cells. (**D**) Representative images of coomassie blue staining of the purified recombinant protein. (**E**) Immunoprecipitation and western blotting analyzing the interaction of STING with endogenous CAD, TBK1 and IRF3 in HCT116 cells. (**F**) Immunoprecipitation and western blotting analyzing the interaction of CAD with endogenous STING, TBK1 and IRF3 in HT29 cells. (**G**) Immunoprecipitation and western blotting analyzing the interaction between wild type and different mutants of STING with CAD (left panel) or the interaction between wild type and different mutants of CAD with STING (right panel).

**Supplemental Figure 2. CAD impairs STING signaling.** (**A**) Western blotting analyses of CAD expression in intestinal epithelial cells and different CRC cell lines. (**B-C**) Western blotting analyses validating the stable cell lines construction in HCT116, HT29 (**B**) and MC38 (**C**) cells. (**D**) Activity of the IFN-β luciferase reporter in the indicated cells at 24 h after irradiation. (**E-F**) Western blotting analyses of p-TBK1, TBK1, p-IRF3 and IRF3 levels in the CAD-KO1, overexpression and the corresponding vector control HCT116 and HT29 cells at 24 h after irradiation. (**G**) Western blotting analysis of phosphorylation of Tbk1 and Irf3, Tbk1 and Irf3 levels in sgVec control and Cad-KO MC38 cells at 24 h after irradiation. (**H**) qPCR analyses of Il-6, Il-8, Ccl5, Cxcl10, Ifn-β, and Tnf-α mRNA in indicated MC38 cells at 24 h after irradiation. (**I**) qPCR analyses of IL-6, IL-8, CCL5, CXCL10, IFN-β, and TNF-α mRNA in the indicated cells at 24 h after irradiation. (**J**) Western blotting analyses of p-TBK1, TBK1, p-IRF3 and IRF3 levels in the indicated cells treated with cGAMP or the vehicle DMSO. (**K-L**) qPCR analyses of CCL5, CXCL10, and IFN-β mRNA in the indicated cells treated with cGAMP or DMSO. (**M**) Western blotting analyses of the IRF3 level in the nuclear and cytoplasmic fractions of the indicated cells at 24 h after irradiation. Lamin B1 and GAPDH served as nuclear and cytoplasmic loading control respectively. Error bars represent mean ± SD. *P* values were determined using two tailed, unpaired t-test (**D**, **H**, **I**, **K** and **L**). *, *P* < 0.05; **, *P* < 0.01; ***, *P* < 0.001; ns, not significant.

**Supplemental Figure 3. CAD acts downstream of cGAS and inhibition of CAD does not interrupt activation of cGAS.**

(**A**, **C**) Western blotting analyses of p-TBK1, TBK1, p-IRF3, and IRF3 levels in indicated HCT116 (**A**) and MC38 (**C**) cells at 24 h after treatment of irradiation plus DMSO or H-151. (**B**, **D**) qPCR analyses of CCL5, CXCL10, and IFN-β mRNA in indicated HCT116 (**B**) and MC38 (**D**) cells at 24 h after treatment of irradiation plus DMSO or H-151. (**E**) Western blotting analyses of p-TBK1, TBK1, p-IRF3, and IRF3 levels in HCT116 cells at 24 h after treatment of irradiation plus DMSO or RU.521. (**F**) qPCR analyses of CCL5, CXCL10, and IFN-β mRNA in HCT116 cells at 24 h after treatment of irradiation plus DMSO or RU.521. (**G**) Immunoprecipitation and western blotting analyses of the effects of CAD on cGAS dimerization in HEK293T cells. (**H**) Cellular cGAMP analyzed by ELISA assay of the indicated cells. Error bars represent mean ± SD. (**I**) Western blotting analyses of p-TBK1, TBK1, p-IRF3 and IRF3 levels in control cells and the CAD-KO1 cells that reintroduced the wild type (WT) or nuclease activity dead mutant (H260A) of CAD at 24 h after irradiation. (**J**) qPCR analyses of CCL5, CXCL10, and IFN-β mRNA in indicated cells at 24 h after irradiation. (**K, L**) Western blotting analyses of p-TBK1, TBK1, p-IRF3 and IRF3 levels in the indicated cells at 24 h after irradiation. (**M, N**) qPCR analyses of CCL5, CXCL10, and IFN-β mRNA in indicated cells at 24 h after irradiation. Error bars represent mean ± SD.*P* values were determined using two tailed, unpaired t-test (**B**, **D**, **F**, **H, J and M**). *, *P* < 0.05; **, *P* < 0.01; ***, *P* < 0.001; ns, not significant.

**Supplemental Figure 4. ICAD does not interact with STING.** (**A**) Co-IP and western blotting assays analyzing the interaction between ICAD and CAD. (**B-C**) Co-IP and western blotting assays analyzing the interaction between ICAD/Icad and STING/Sting in 293T (**B**) and MC38 (**C**) cells.

**Supplemental Figure 5. Inhibition of CAD reprograms tumor immunomicroenviroment after irradiation.**

(**A**) T cell killing efficiency of the indicated MC38 cells treated with irradiation. Each spot intensity represents the live cancer cell quantity and the relative fold ratio of surviving cells is shown. (**B**) Representative images of Flow Cytometry Gating strategy. (**C-D**) Quantification of intratumoral DC (CD86^+^MHCII^+^, A) and MDSC (CD11b^+^Gr1^+^, B) cells among CD45^+^ cells. (**E**) Representative images and quantification of IHC staining of CD8 of the unirradiated abscopal tumors in mice. Scale bars, 100 μm. (**F**) Schematic diagrams of subcutaneous tumor models and the treatment related to Fig. 6H. Error bars represent mean ± SD. *P* values were determined using two tailed, unpaired t-test (**A** and **E**) or one-way ANOVA with Tukey’s multiple comparison test (**C** and **D**). *, *P* < 0.05; **, *P* < 0.01; ***, *P* < 0.001; ns, not significant.
