# Supplementary figures and images for: CAD hijacks STING to impair antitumor immunity and radiotherapy efficacy of colorectal cancer
Source: Cell Death Dis. 2025 Aug 23;16(1):641. doi: 10.1038/s41419-025-07964-8 (PMC12375107; doi:10.1038/s41419-025-07964-8)

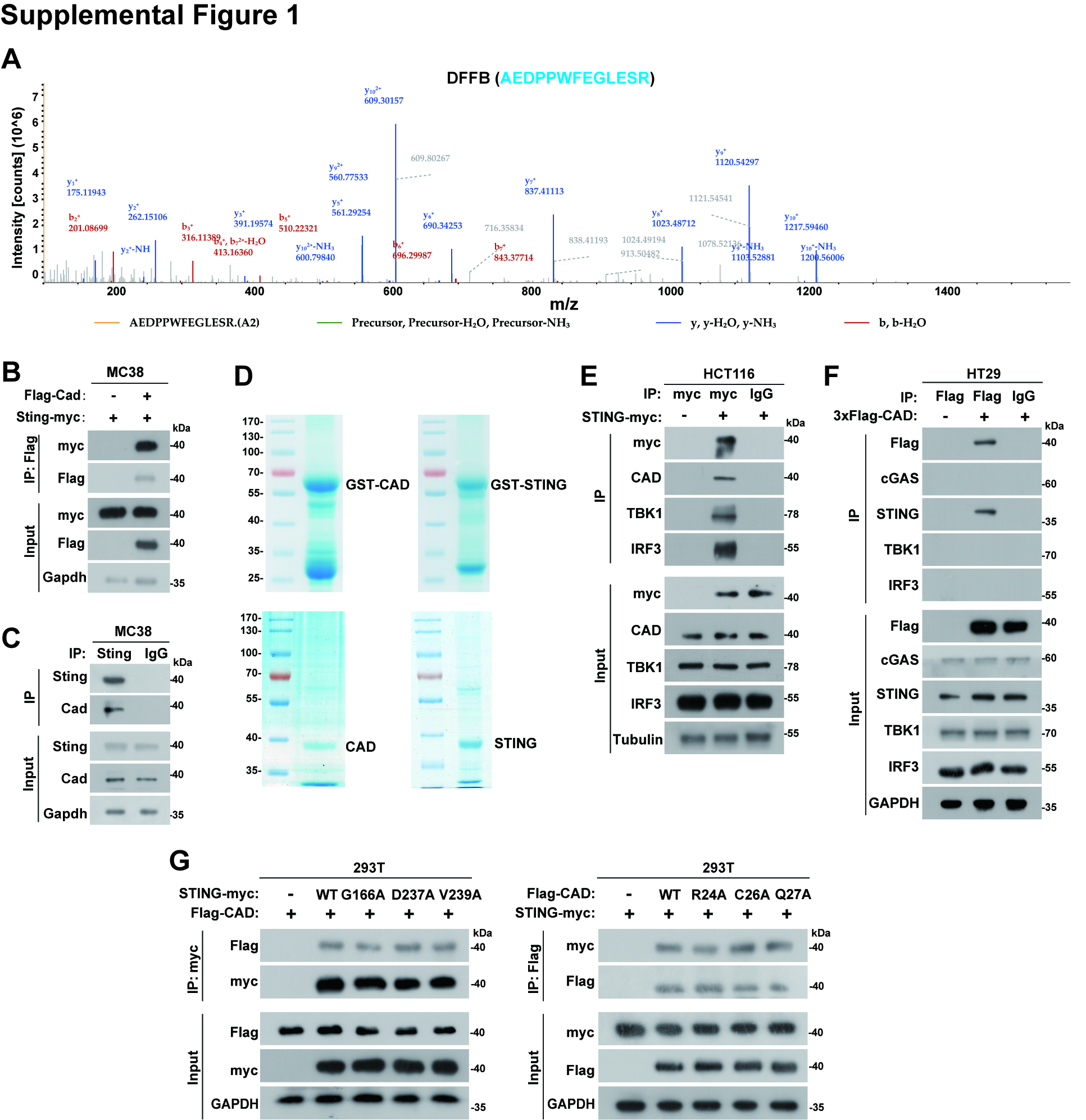

Supplement: Supplementary file 2 — Supplemental Figure 1 [file 41419_2025_7964_MOESM2_ESM.tif]

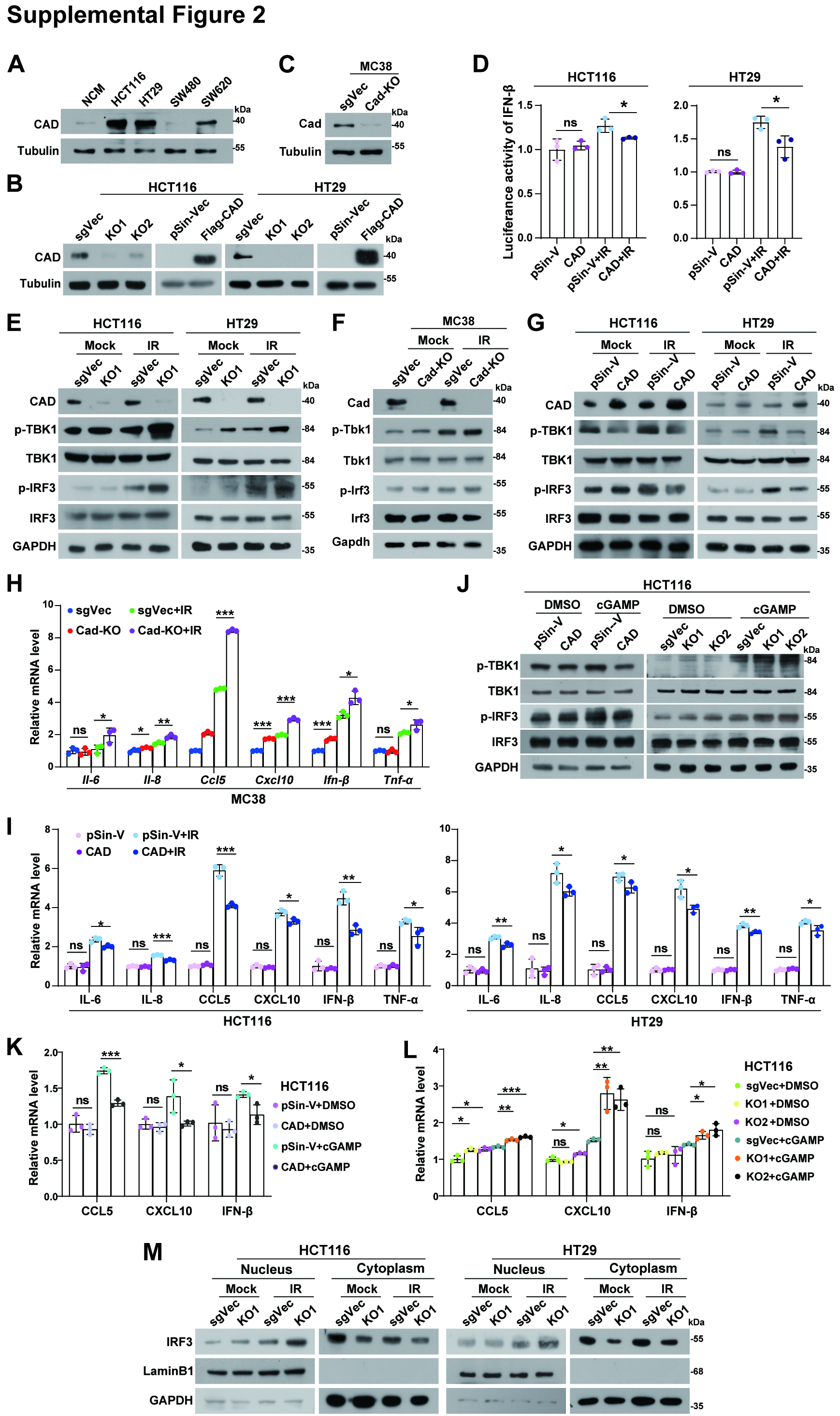

Supplement: Supplementary file 3 — Supplemental Figure 2 [file 41419_2025_7964_MOESM3_ESM.tif]

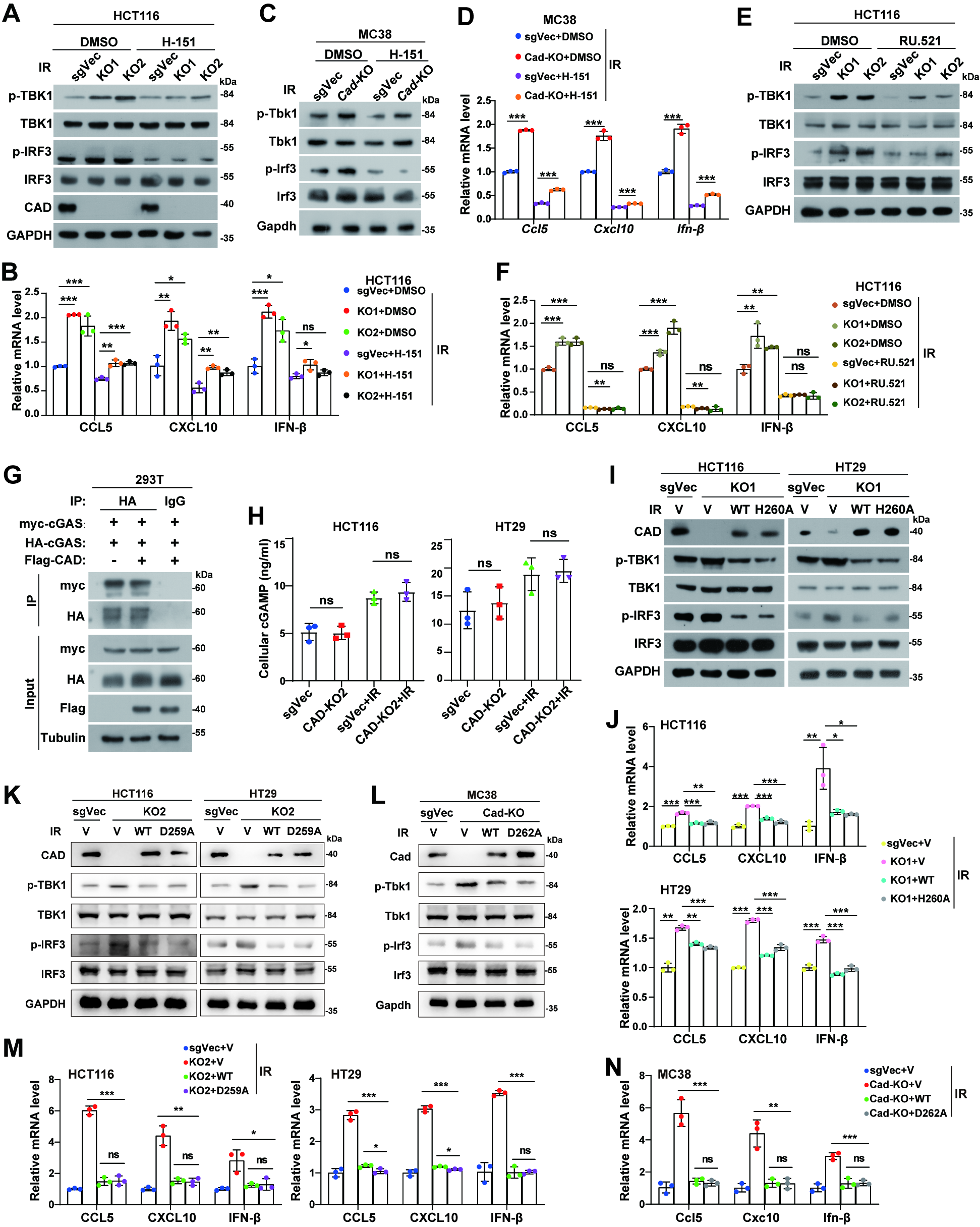

Supplement: Supplementary file 4 — Supplemental Figure 3 [file 41419_2025_7964_MOESM4_ESM.tif]

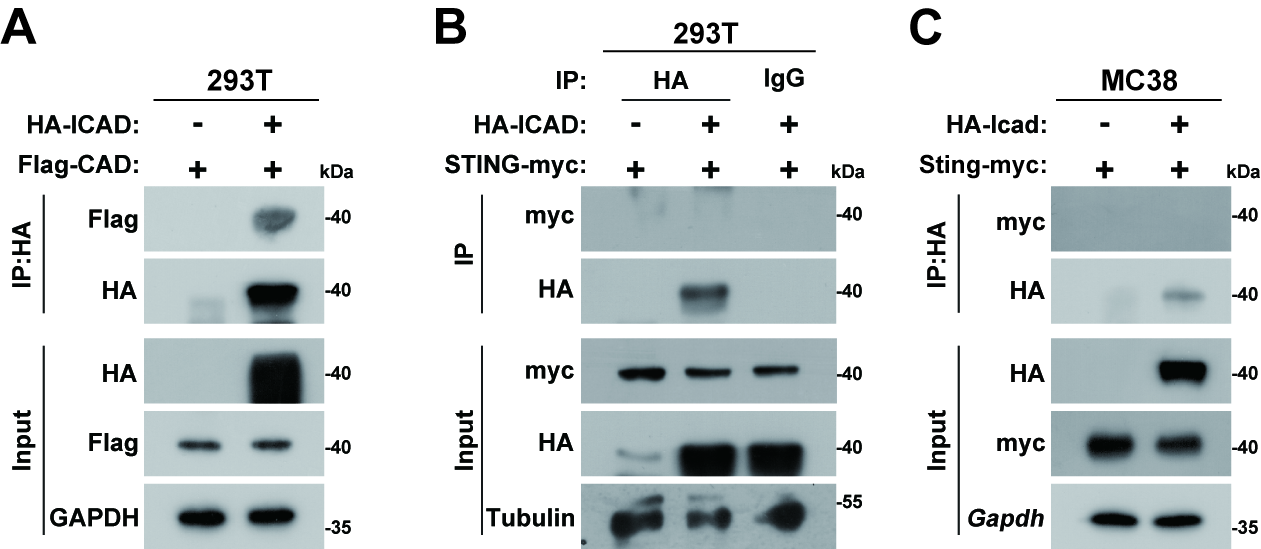

Supplement: Supplementary file 5 — Supplemental Figure 4 [file 41419_2025_7964_MOESM5_ESM.tif]

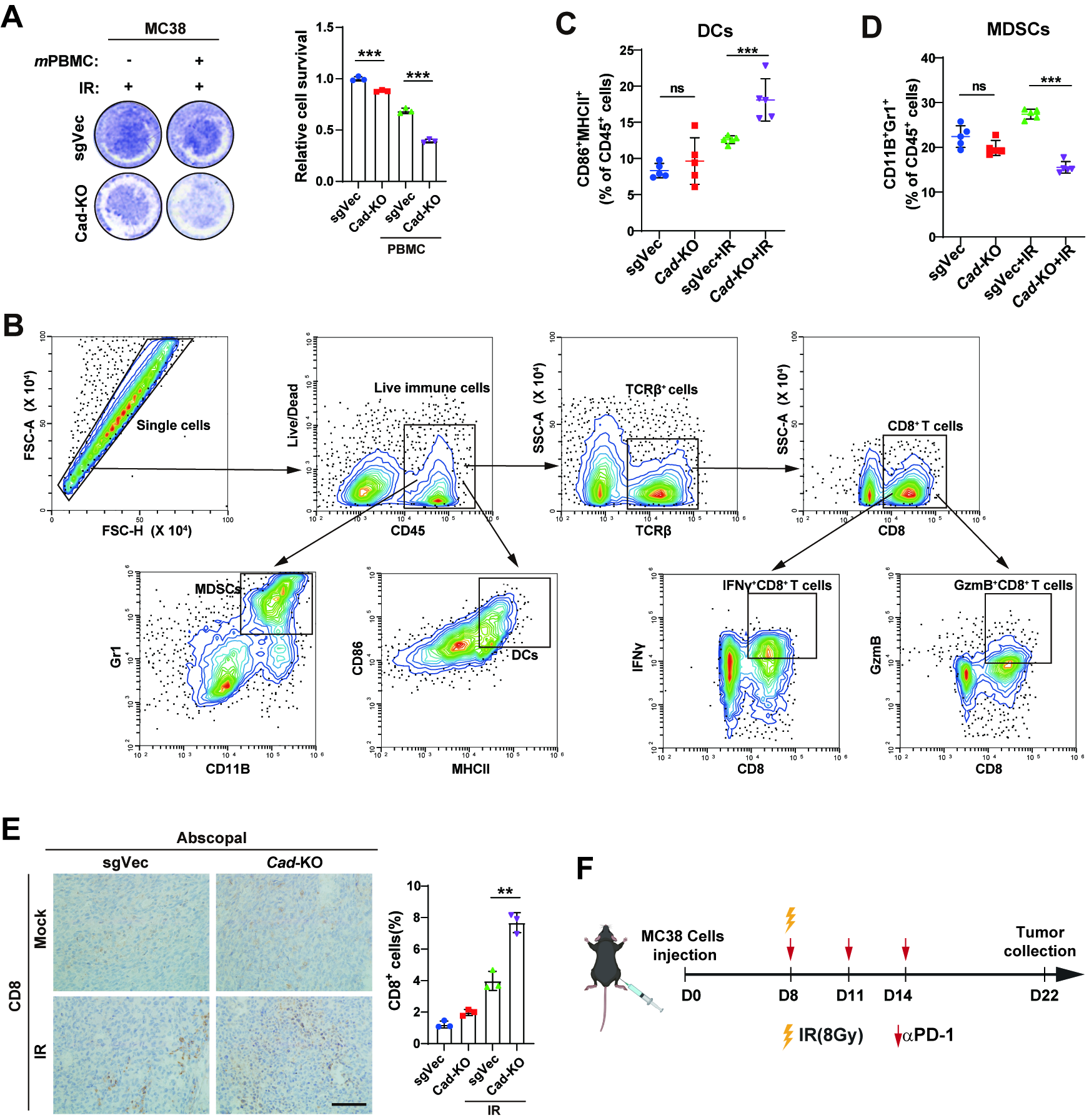

Supplement: Supplementary file 6 — Supplemental Figure 5 [file 41419_2025_7964_MOESM6_ESM.tif]
