# Supplementary material for: CAD hijacks STING to impair antitumor immunity and radiotherapy efficacy of colorectal cancer
Source: Cell Death Dis. 2025 Aug 23;16(1):641. doi: 10.1038/s41419-025-07964-8 (PMC12375107; doi:10.1038/s41419-025-07964-8)
Supplement: Supplementary file 7 — Uncropped western blots [file 41419_2025_7964_MOESM7_ESM.pdf]

Figure 1C

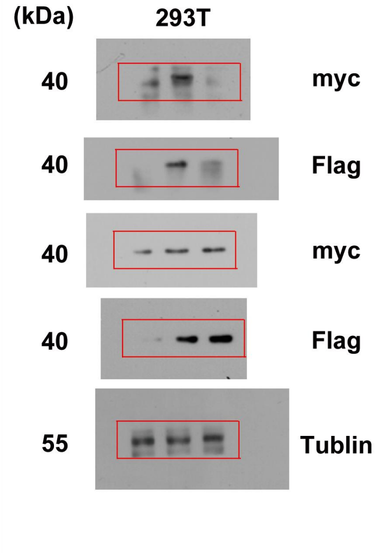

Figure 1D

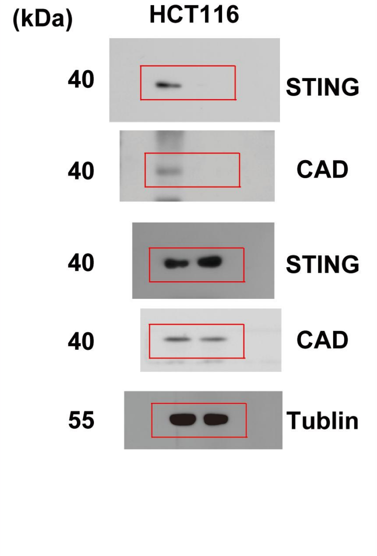

Figure 1G

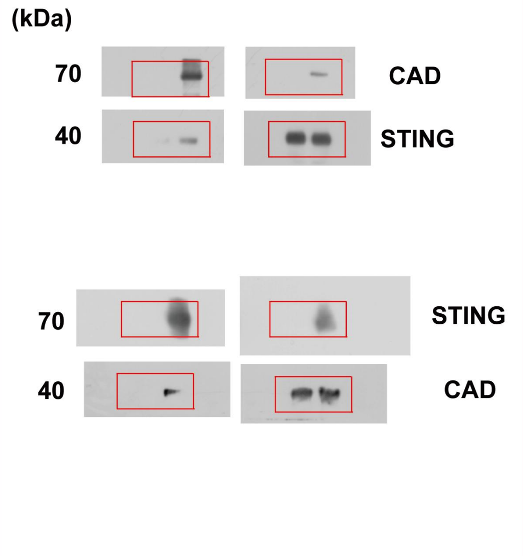

Figure 1H

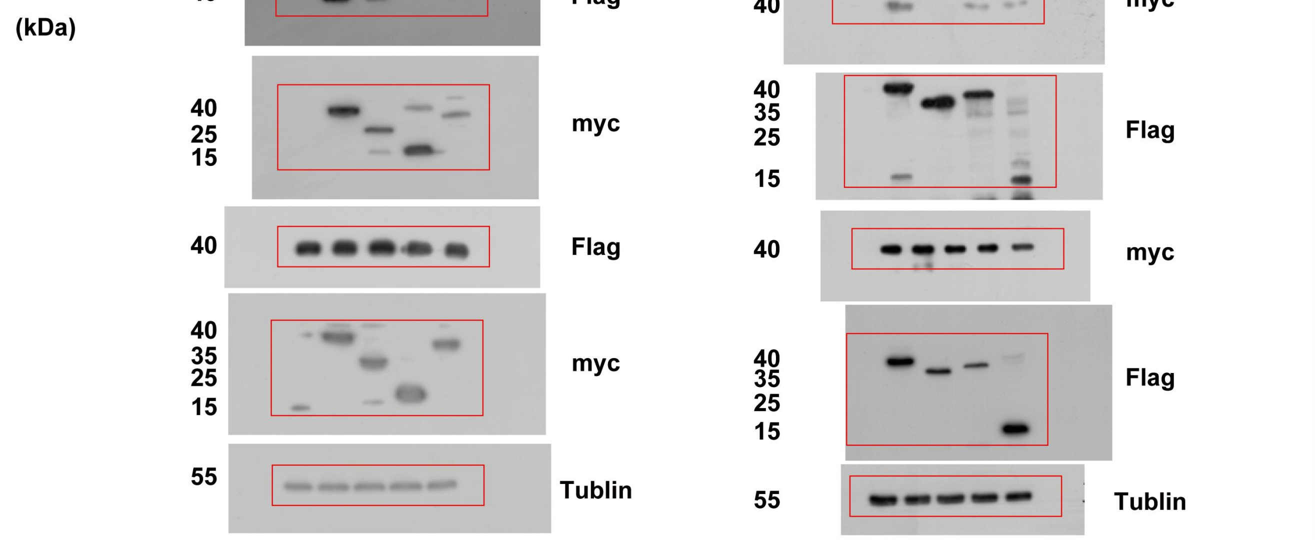

Figure 1J

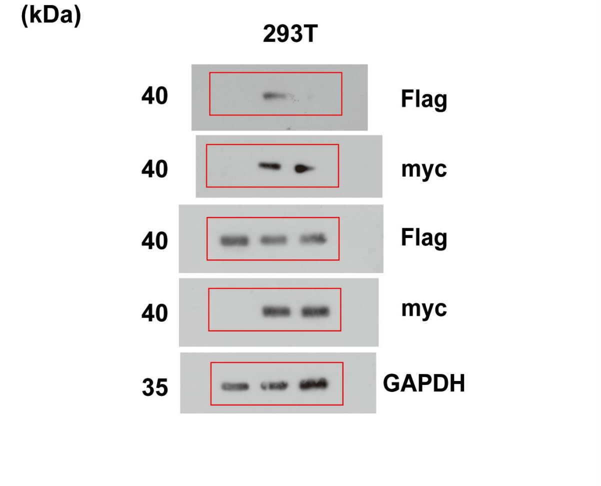

Figure 1K

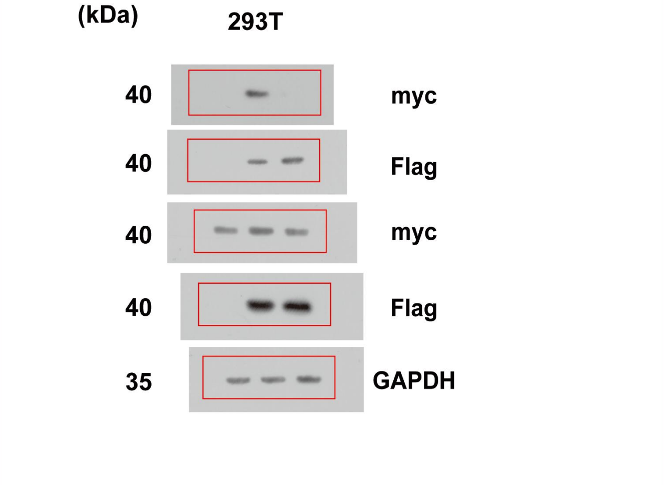

Supplemental Figure 1B

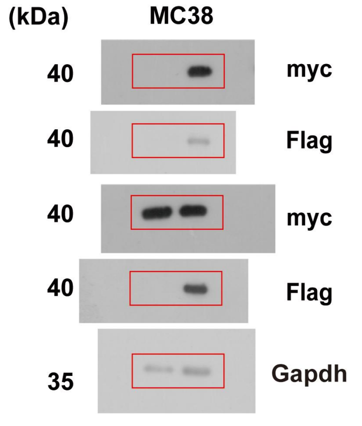

Supplemental Figure 1C

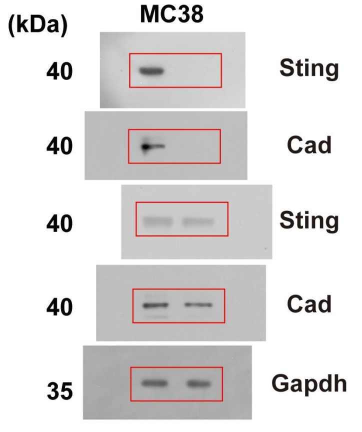

Supplemental Figure 1D

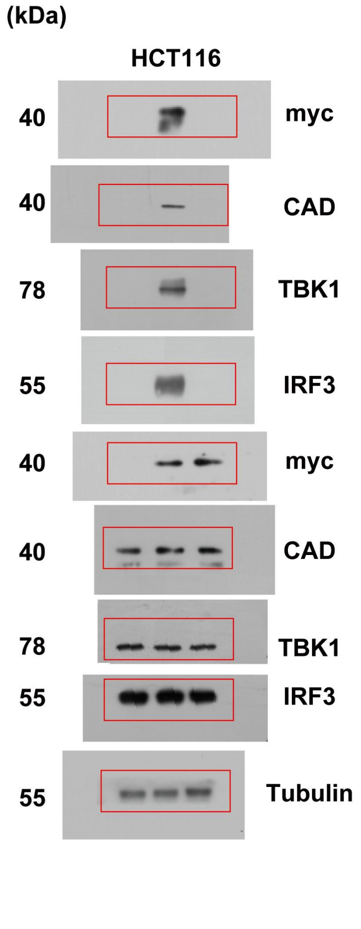

Supplemental Figure 1E

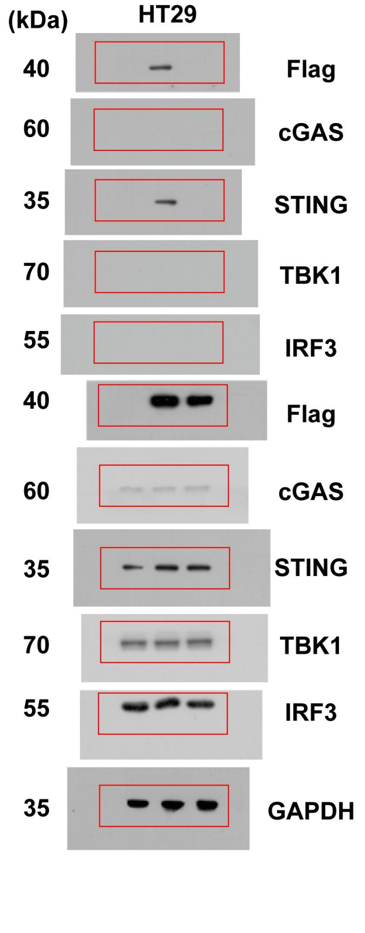

Supplemental Figure 1G

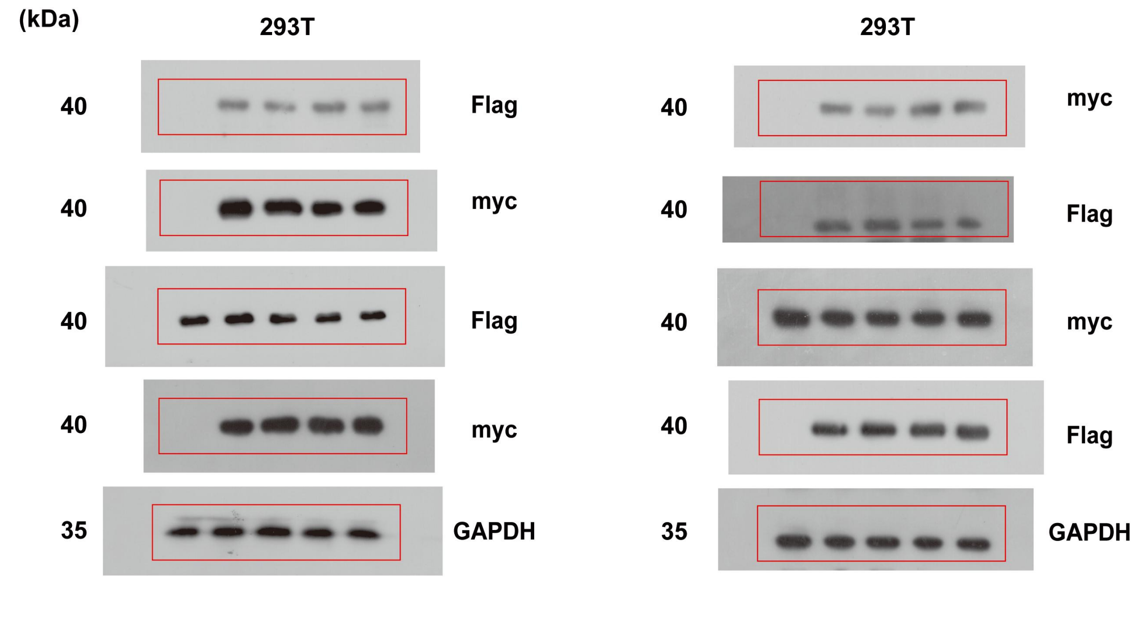

Figure 2F

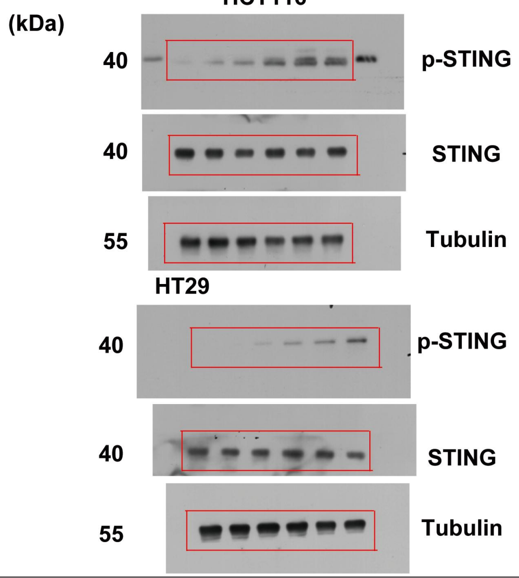

Figure 2G

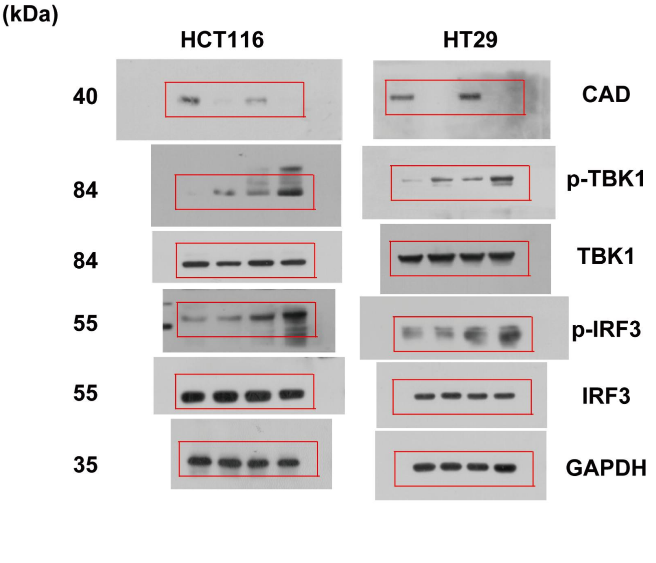

Figure 2I

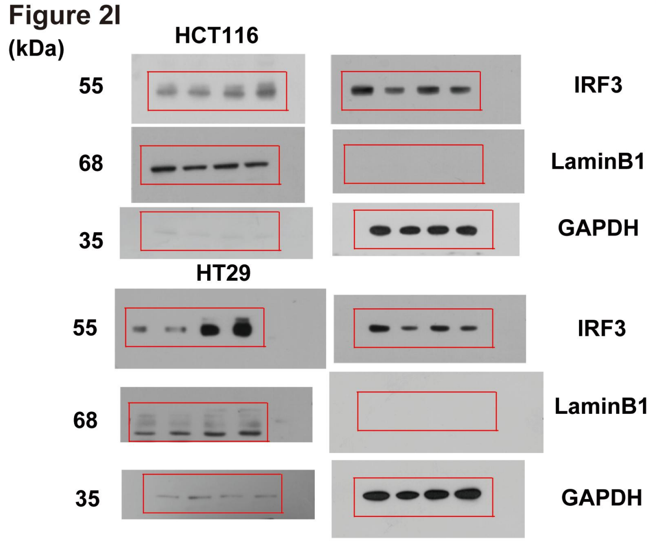

Supplemental Figure 2A

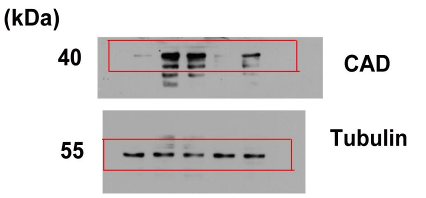

Supplemental Figure 2C

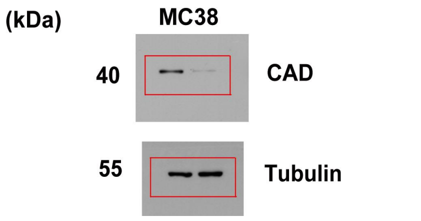

Supplemental Figure 2B

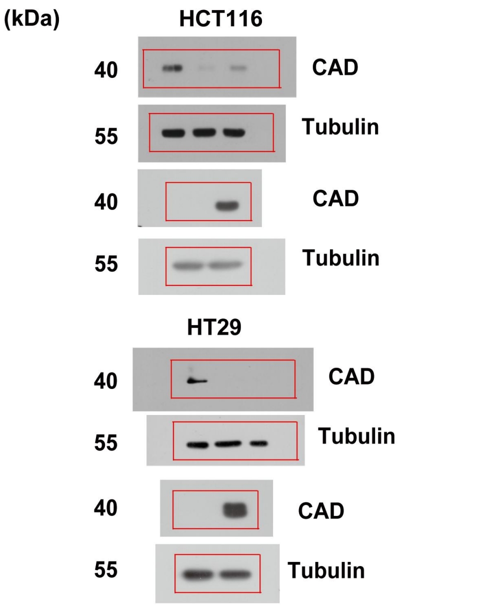

Supplemental Figure 2E

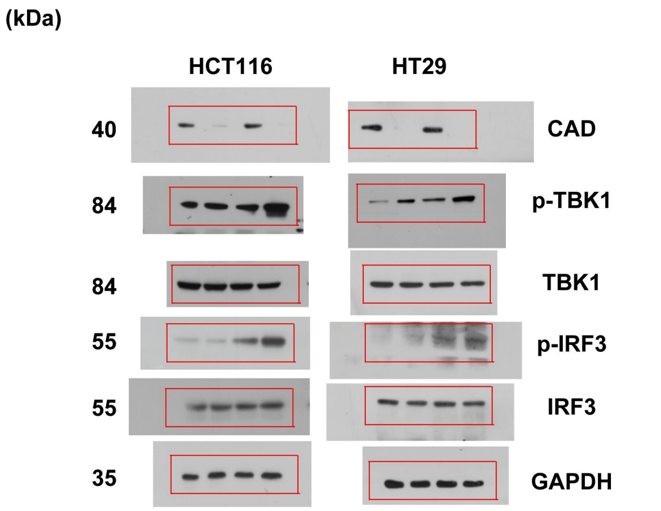

Supplemental Figure 2F

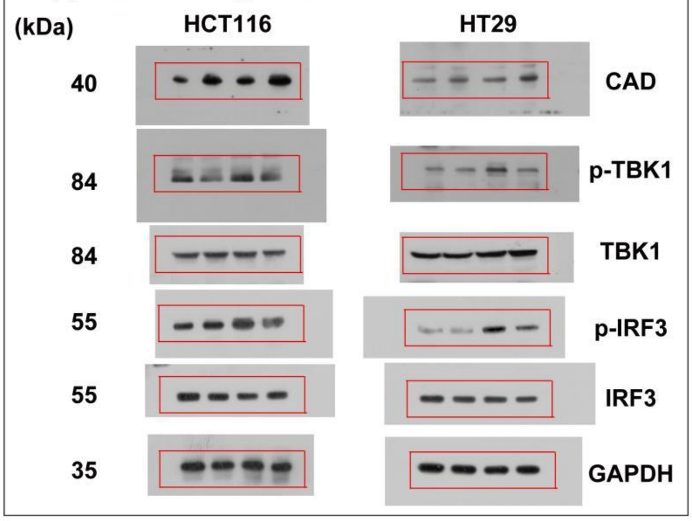

Supplemental Figure 2G

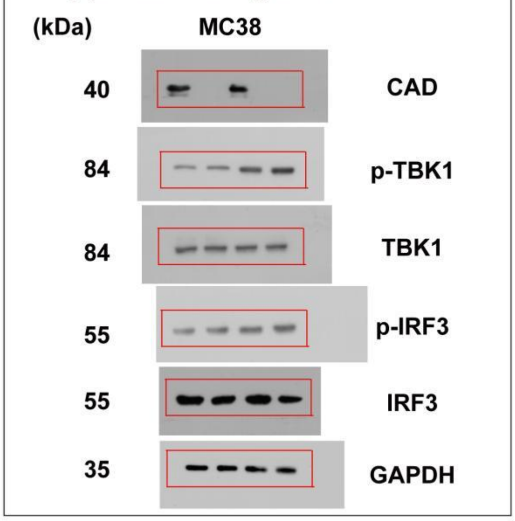

Supplemental Figure 2J

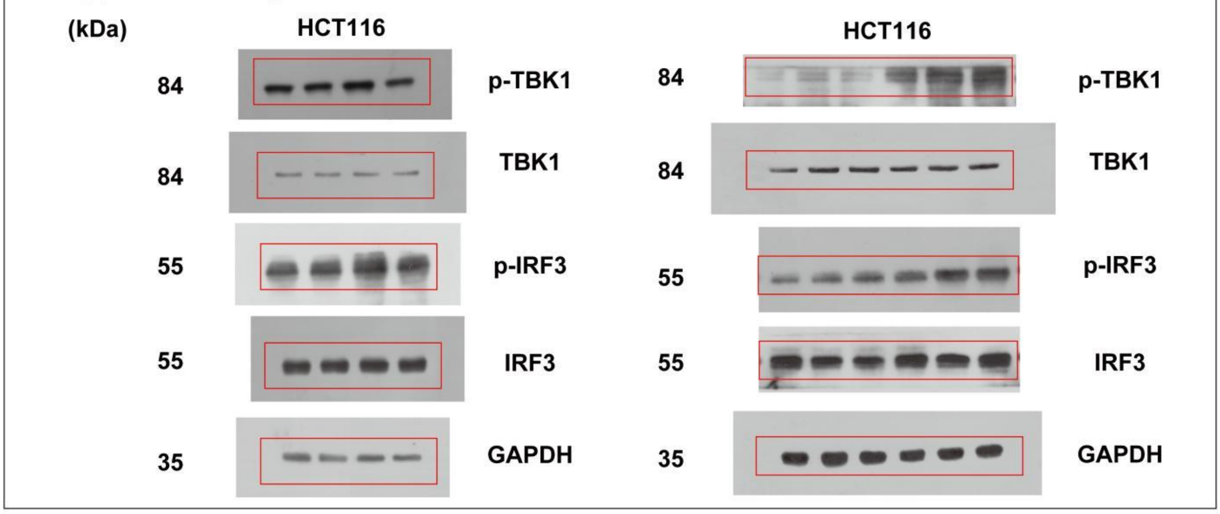

Supplemental Figure 2M

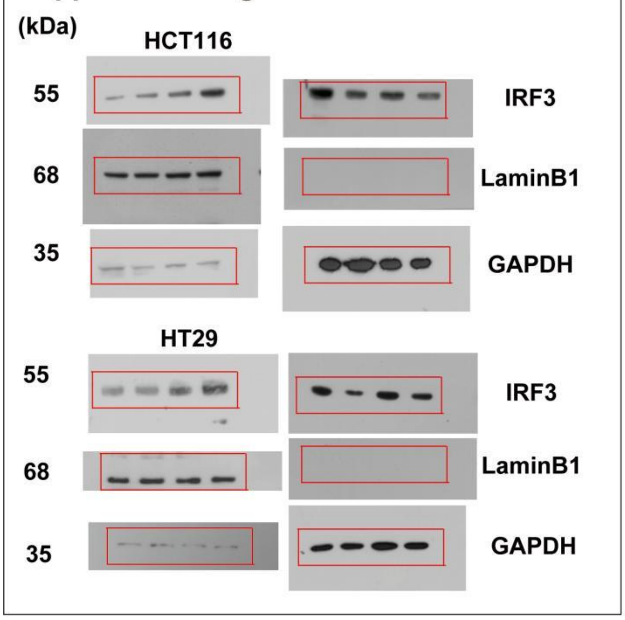

Figure 3A

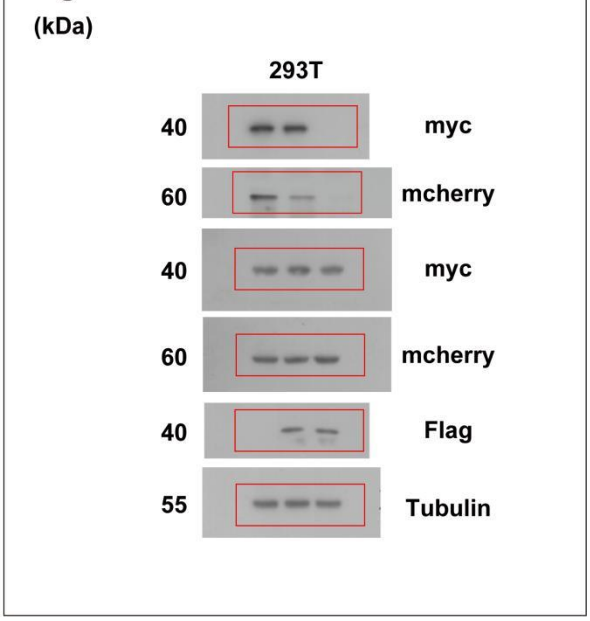

Figure 3B

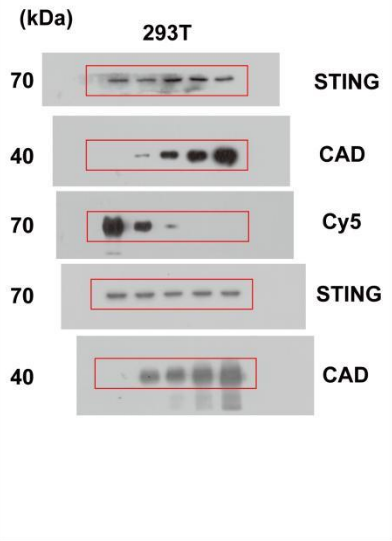

Figure 3E

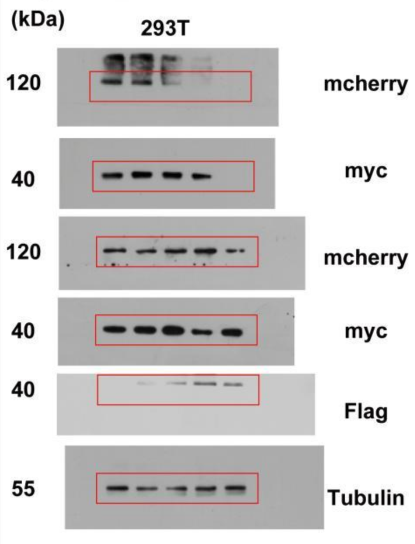

Figure 3F

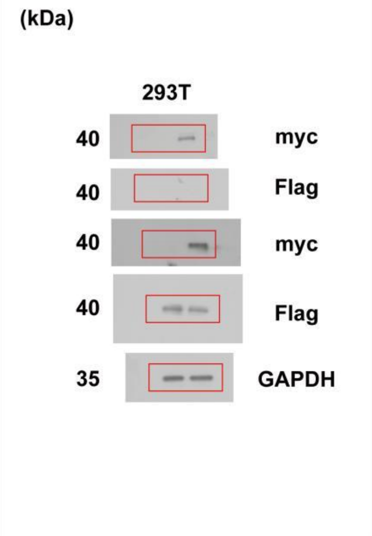

Figure 3G

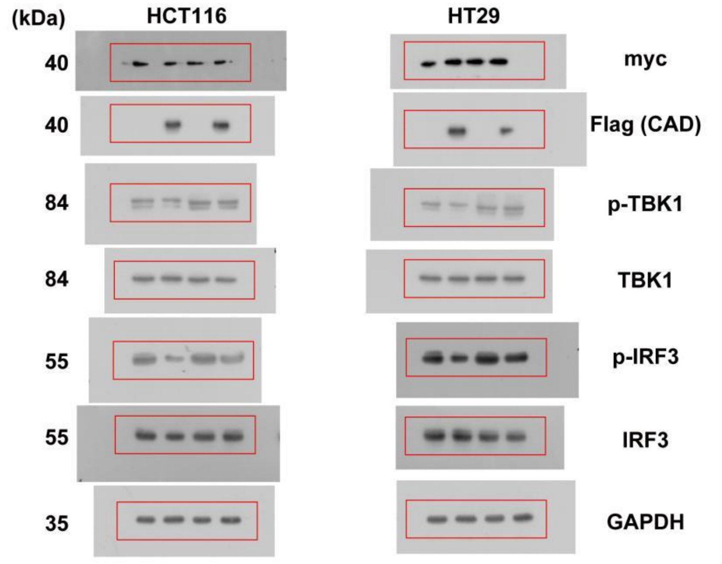

Figure 3H

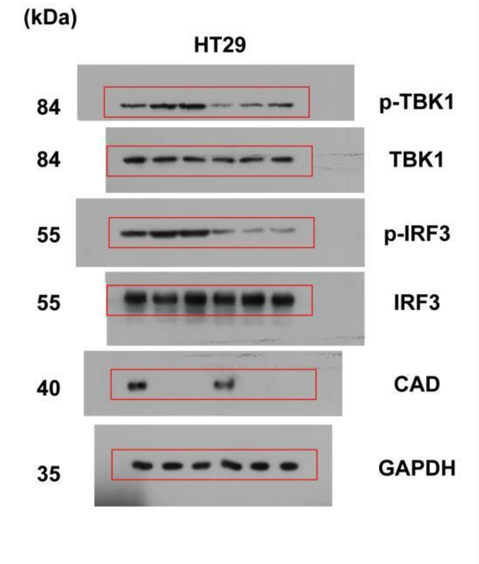

Figure 3J

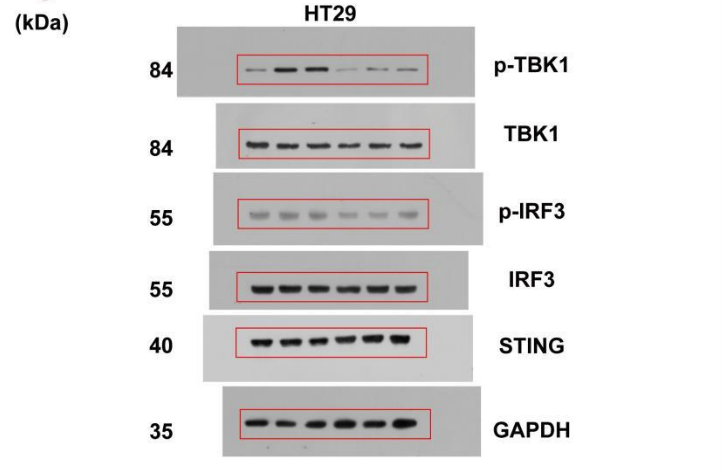

Figure 3L

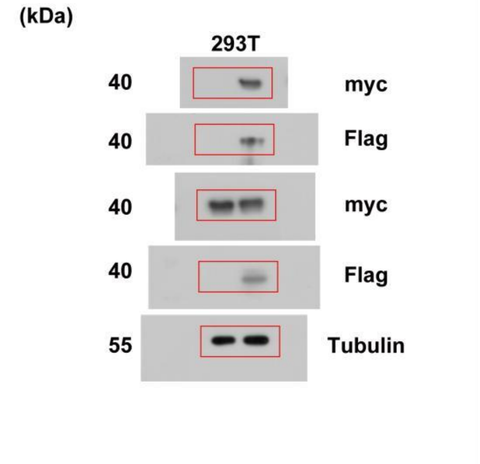

Figure 3M

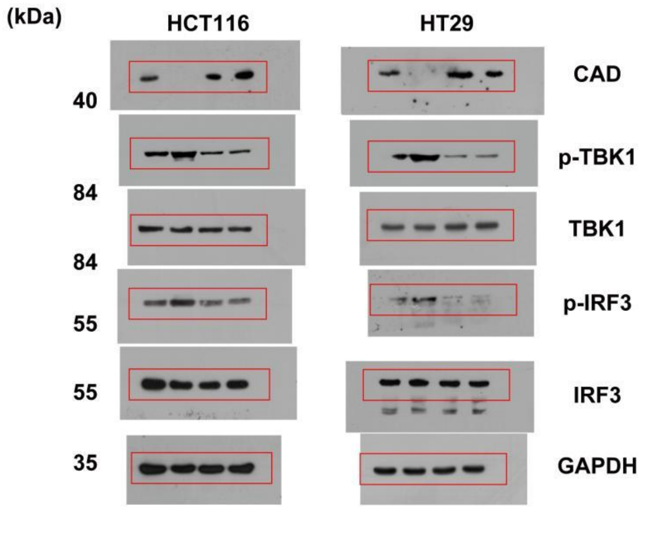

Supplemental Figure 3A

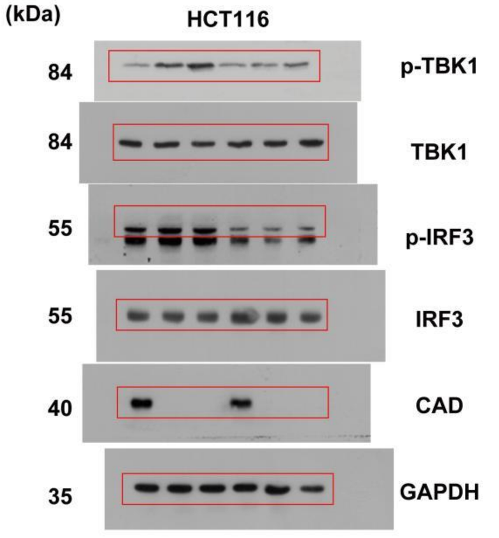

Supplemental Figure 3C

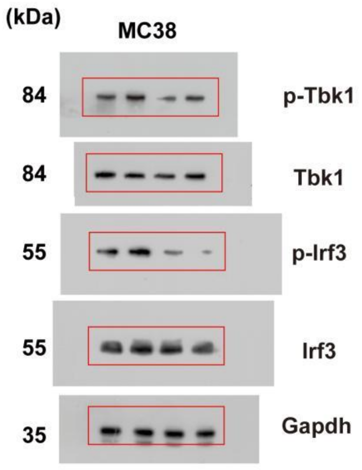

Supplemental Figure 3E

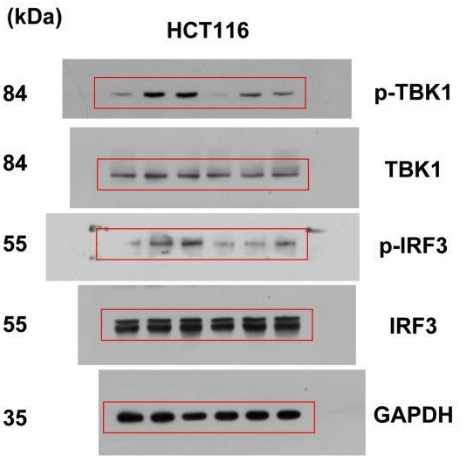

Supplemental Figure 3G

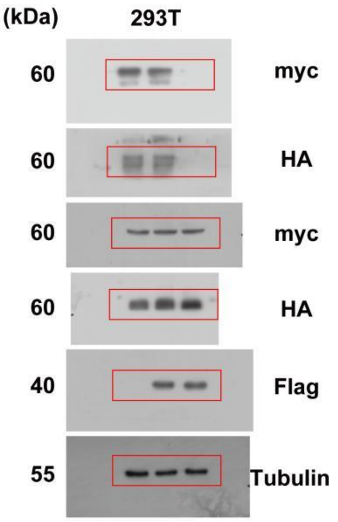

Supplemental Figure 3I

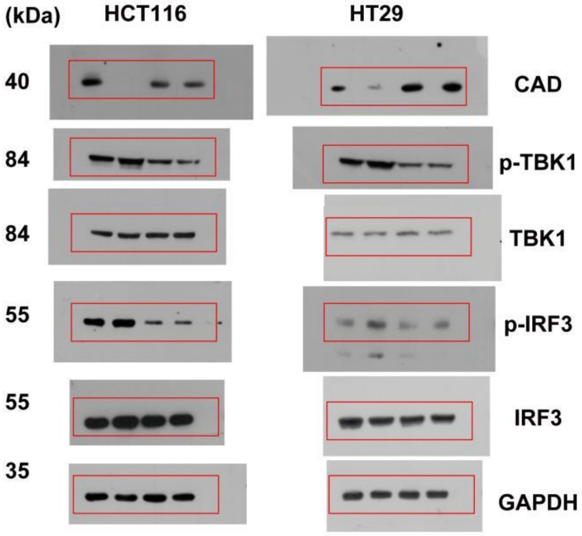

Supplemental Figure 3K

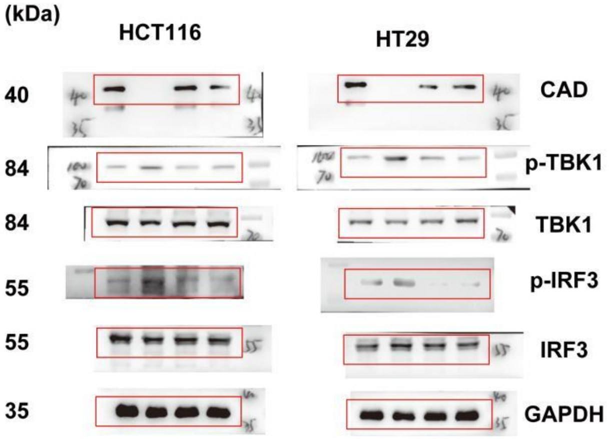

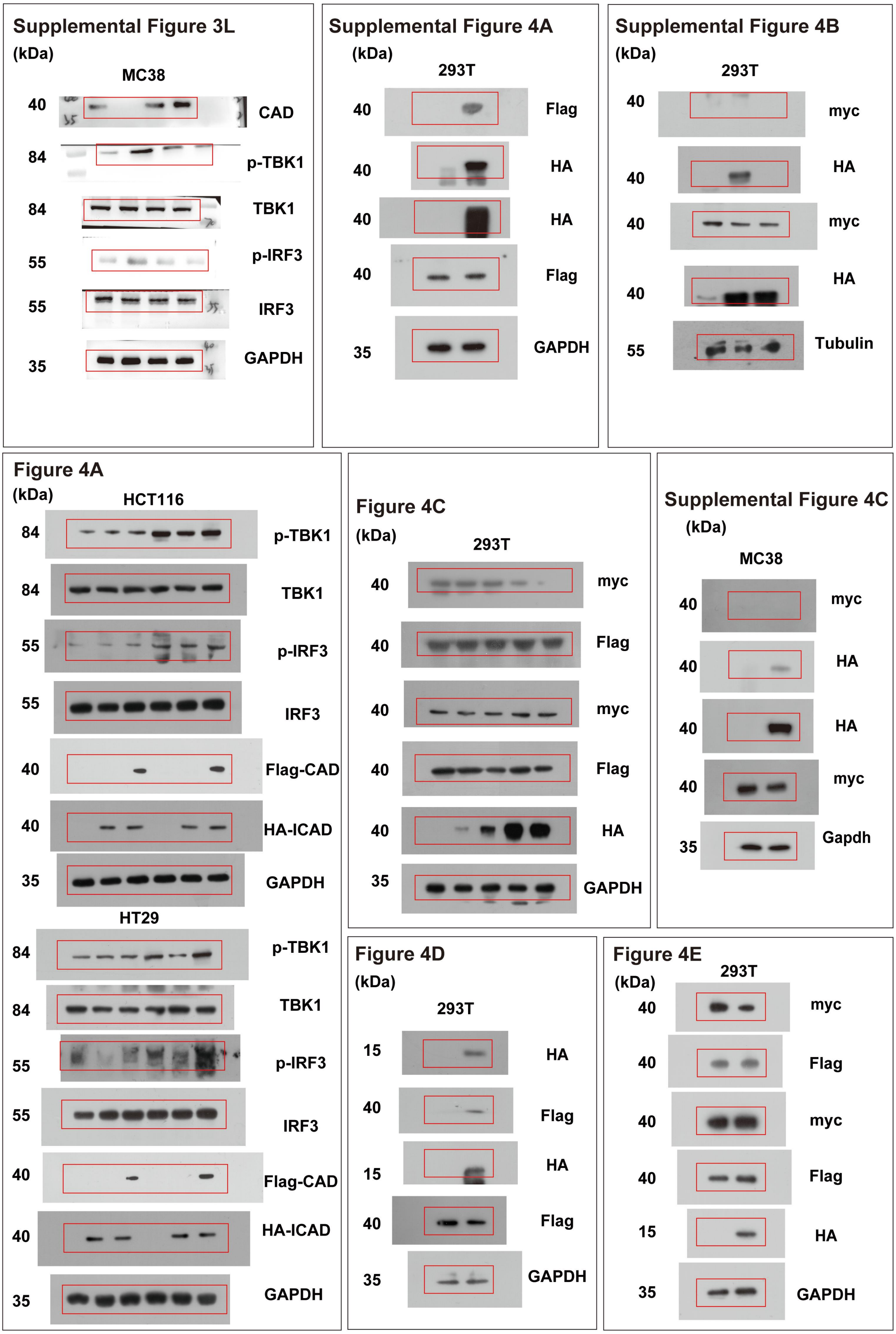
Supplemental Figure 3L
(kDa)
MC38
40 CAD
84 p-TBK1
84 TBK1
55 p-IRF3
55 IRF3
35 GAPDH
Supplemental Figure 4A
(kDa)
293T
40 Flag
40 HA
40 HA
40 Flag
35 GAPDH
Supplemental Figure 4B
(kDa)
293T
40 myc
40 HA
40 myc
40 HA
55 Tubulin
Figure 4A
(kDa)
HCT116
84 p-TBK1
84 TBK1
55 p-IRF3
55 IRF3
40 Flag-CAD
40 HA-ICAD
35 GAPDH
HT29
84 p-TBK1
84 TBK1
55 p-IRF3
55 IRF3
40 Flag-CAD
40 HA-ICAD
35 GAPDH
Figure 4C
(kDa)
293T
40 myc
40 Flag
40 myc
40 Flag
40 HA
35 GAPDH
Supplemental Figure 4C
(kDa)
MC38
40 myc
40 HA
40 HA
40 myc
35 Gapdh
Figure 4D
(kDa)
293T
15 HA
40 Flag
15 HA
40 Flag
35 GAPDH
Figure 4E
(kDa)
293T
40 myc
40 Flag
40 myc
40 Flag
15 HA
35 GAPDH
